# Host-derived apolipoproteins play comparable roles with viral secretory proteins E^rns^ and NS1 in the infectious particle formation of *Flaviviridae*

**DOI:** 10.1371/journal.ppat.1006475

**Published:** 2017-06-23

**Authors:** Takasuke Fukuhara, Tomokazu Tamura, Chikako Ono, Mai Shiokawa, Hiroyuki Mori, Kentaro Uemura, Satomi Yamamoto, Takeshi Kurihara, Toru Okamoto, Ryosuke Suzuki, Kentaro Yoshii, Takeshi Kurosu, Manabu Igarashi, Hiroshi Aoki, Yoshihiro Sakoda, Yoshiharu Matsuura

**Affiliations:** 1 Department of Molecular Virology, Research Institute for Microbial Diseases, Osaka University, Osaka, Japan; 2 Laboratory of Microbiology, Department of Disease Control, Graduate School of Veterinary Medicine, Hokkaido University, Hokkaido, Japan; 3 School of Veterinary Nursing and Technology, Faculty of Veterinary Science, Nippon Veterinary and Life Science University, Tokyo, Japan; 4 Department of Virology II, National Institute of Infectious Diseases, Tokyo, Japan; 5 Laboratory of Public Health, Department of Environmental Veterinary Sciences, Graduate School of Veterinary Medicine, Hokkaido University, Hokkaido, Japan; 6 Department of Virology I, National Institute of Infectious Diseases, Tokyo, Japan; 7 Global Institution for Collaborative Research and Education (GI-CoRE), Hokkaido University, Hokkaido, Japan; 8 Division of Global Epidemiology, Research Center for Zoonosis Control, Hokkaido University, Hokkaido, Japan; The University of Chicago, UNITED STATES

## Abstract

Amphipathic α-helices of exchangeable apolipoproteins have shown to play crucial roles in the formation of infectious hepatitis C virus (HCV) particles through the interaction with viral particles. Among the *Flaviviridae* members, pestivirus and flavivirus possess a viral structural protein E^rns^ or a non-structural protein 1 (NS1) as secretory glycoproteins, respectively, while *Hepacivirus* including HCV has no secretory glycoprotein. In case of pestivirus replication, the C-terminal long amphipathic α-helices of E^rns^ are important for anchoring to viral membrane. Here we show that host-derived apolipoproteins play functional roles similar to those of virally encoded E^rns^ and NS1 in the formation of infectious particles. We examined whether E^rns^ and NS1 could compensate for the role of apolipoproteins in particle formation of HCV in apolipoprotein B (ApoB) and ApoE double-knockout Huh7 (BE-KO), and non-hepatic 293T cells. We found that exogenous expression of either E^rns^ or NS1 rescued infectious particle formation of HCV in the BE-KO and 293T cells. In addition, expression of apolipoproteins or NS1 partially rescued the production of infectious pestivirus particles in cells upon electroporation with an E^rns^-deleted non-infectious RNA. As with exchangeable apolipoproteins, the C-terminal amphipathic α-helices of E^rns^ play the functional roles in the formation of infectious HCV or pestivirus particles. These results strongly suggest that the host- and virus-derived secretory glycoproteins have overlapping roles in the viral life cycle of *Flaviviridae*, especially in the maturation of infectious particles, while E^rns^ and NS1 also participate in replication complex formation and viral entry, respectively. Considering the abundant hepatic expression and liver-specific propagation of these apolipoproteins, HCV might have evolved to utilize them in the formation of infectious particles through deletion of a secretory viral glycoprotein gene.

## Author summary

The family *Flaviviridae* consists of 4 genera, namely *Flavivirus*, *Pestivirus*, *Pegivirus*, and *Hepacivirus*. Flaviviruses and pestiviruses can infect various species and tissues; however, infection of pegivirus and hepacivirus is observed in a strikingly restricted range of tissue and hosts. Although all the *Flaviviridae* viruses possess a similar genome structure, hepatitis C virus (HCV) from *Hepacivirus* encodes no secretory glycoprotein, such as E^rns^ of pestivirus and NS1 of flavivirus. The apolipoproteins, one of the host secretory glycoproteins, play important roles in the formation of infectious HCV particles through the interaction with viral particles. The data presented here show that the host-derived apolipoproteins and viral-derived E^rns^ and NS1 have overlapping roles in the maturation of infectious particles of *Flaviviridae*. Considering an abundant expression of apolipoproteins in the liver and their liver-specific propagation, HCV might have evolved to utilize the apolipoproteins in the formation of infectious particles through deletion of a gene encoding a secretory viral glycoprotein. The data of this manuscript also suggest that utilization of host factors in the viral life cycle is closely associated with the tissue- and species-specificities and evolution among *Flaviviridae* viruses.

## Introduction

The family *Flaviviridae* consists of 4 genera, *Flavivirus*, *Pestivirus*, *Pegivirus*, and *Hepacivirus*. *Flaviviridae* viruses commonly possess a single-stranded positive-sense RNA encoding 2–4 structural proteins and 7–8 non-structural proteins [1]. The host range and tissue tropism of these proteins are strikingly different. The genus *Flavivirus* is composed of more than 50 species with a wide host range from reptiles to mammals; these mosquito- or tick-borne viruses include dengue virus (DENV), Japanese encephalitis virus (JEV), and tick-borne encephalitis virus (TBEV) [2]. The genus *Pestivirus* comprises economically important pathogens of even-toed ungulate animals, including classical swine fever virus (CSFV), bovine viral diarrhea virus (BVDV), and border disease virus (BDV), which cause major losses in livestock farming [3]. In the case of hepatitis C virus (HCV) from the genus *Hepacivirus*, the species-specificity is restricted to humans and chimpanzees [4].

Although the genome structure of HCV resembles that of pestivirus, a structural protein E^rns^ is missing in HCV [1, 5]. E^rns^ has been shown to be essential for infectious particle formation [5]. Although E^rns^ has no transmembrane domain, the C-terminal amphipathic α-helices are important for anchoring to the membrane of viral particles [5]. Interestingly, the genus *Flavivirus* also encodes a non-structural secretory protein NS1 that is translocated to the lumen of the endoplasmic reticulum (ER) as a dimer or hexamer, and a membrane-binding dimer is known to participate in formation of the viral RNA replication complex [6]. However, the functional role of the hexametric form of NS1 in viral life cycle is not yet well understood. A recent report suggests that NS1 is involved not only in viral RNA replication but also in particle formation [7, 8]. Because HCV does not encode genes for secretory proteins such as E^rns^ and NS1, host factors are suggested to compensate for the role of viral secretory proteins in the life cycle of HCV. Previous studies have shown that exchangeable apolipoproteins including apolipoprotein E (ApoE) participate in the formation of infectious HCV particles [4, 9]. The amphipathic α-helices from the apolipoproteins have been shown to be important for production of the infectious HCV particles through the interaction with viral particles [10]. In addition, we recently reported that a cellular protein, hCAP18/LL-37 (CAMP), that contains amphipathic α-helices can compensate for the role of apolipoproteins in the formation of infectious HCV particles, indicating that expression of secretory membrane-binding proteins such as apolipoproteins and CAMP is essential for the maturation of HCV particles [11]. Because these host factors interact with the ER-derived membrane in a manner similar to E^rns^ and NS1, we hypothesized that the roles of E^rns^ and NS1 in infectious particle formation are similar to those of secretory membrane-binding host factors.

Here, we showed that E^rns^ and NS1 could compensate for the roles of apolipoproteins in the formation of infectious particles of HCV. By using E^rns^-deleted CSFV generated by a reverse genetics system, we revealed that both exchangeable apolipoproteins and NS1 could partially compensate for the roles of E^rns^ in the formation of infectious pestivirus particles. These results suggest that the host- and virus-derived secretory glycoproteins have overlapping roles in the viral life cycle of *Flaviviridae*, especially in maturation of infectious particles, while E^rns^ and NS1 also participate in replication complex formation and viral entry, respectively. HCV might have evolved to utilize host factors including exchangeable apolipoproteins through the loss of a viral gene encoding secretory glycoproteins.

## Results

### E^rns^ can compensate for the role of apolipoproteins in particle formation of HCV

**Fig 1:**
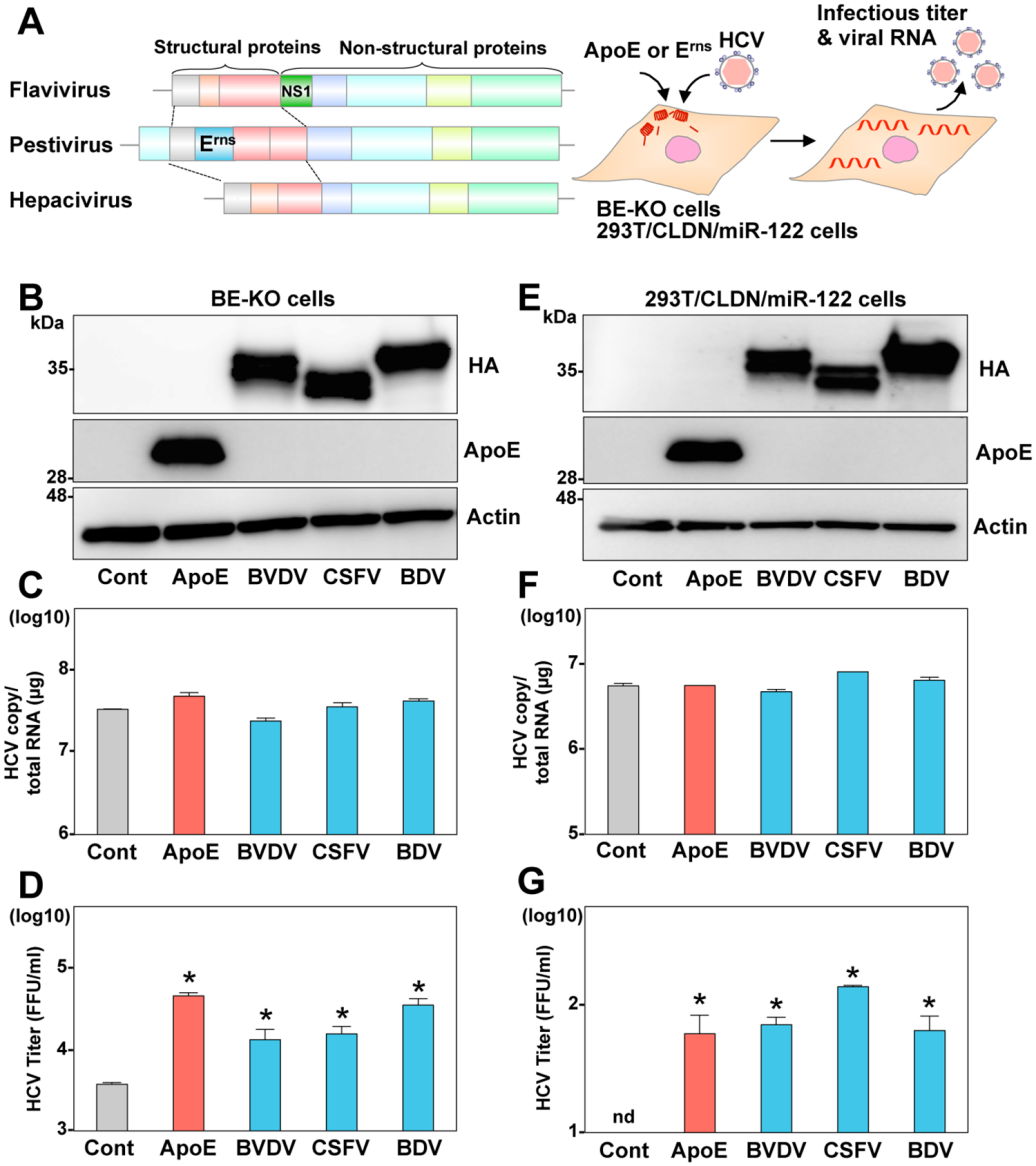
E^rns^ can compensate for the role of apolipoproteins in the infectious particle formation of HCV. (A) Gene structures of flaviviruses, pestiviruses, and hepaciviruses, and the experimental procedure. (B) Expression of ApoE and HA-tagged E^rns^ (HA- E^rns^) from BVDV, CSFV, and BDV was determined by immunoblotting at 48-h post-transduction of lentiviruses into the BE-KO cells. Intracellular HCV RNA (C) and extracellular infectious titers (D) were determined at 72-h post-infection with JFH1 HCV at a multiple of infection (MOI) of 1 by qRT-PCR and focus-forming assay, respectively. (E) Expression of ApoE and HA-E^rns^ in 293T/CLDN1/miR-122 cells was determined by immunoblotting analysis. Intracellular HCV RNA (F) and extracellular infectious titers (G) were determined at 72-h post-infection with JFH1 HCV at an MOI of 10 by qRT-PCR and focus-forming assay, respectively. In all cases, asterisks indicate significant differences (* *p* < 0.01) versus the results of the control cells.

As previously reported, ApoB and ApoE double-knockout Huh7 (BE-KO) cells exhibited an impairment of infectious HCV particle formation, and exogenous expression of ApoE facilitated efficient particle formation (S1 Fig) [10]. To examine whether E^rns^ of pestivirus can compensate for the role of apolipoproteins in the formation of infectious HCV particles, ApoE or HA-tagged E^rns^ (HA-E^rns^) was lentivirally expressed in BE-KO cells and intracellular HCV RNA and infectious viral titers in the supernatants were determined at 72-h post-infection with HCV (Fig 1A–1D). Expression of HA-E^rns^ derived from various pestiviruses, including BVDV, CSFV, and BDV, in BE-KO cells led to a significant recovery of the formation of HCV infectious particles to nearly the level in the control cells, while HCV RNA replication was not affected by the expression of these proteins. In addition, expression of HA-E^rns^ in the BE-KO cells also enhanced infectious titers of Con1/JFH1 chimeric HCV-possessing genotype 1b structural proteins (S2 Fig), suggesting that E^rns^ can compensate for the role of exchangeable apolipoproteins in the formation of infectious particles not only of genotype 2 but also of genotype 1 HCV. Reconstitution of a complete HCV propagation in non-hepatic cells has been accomplished in 293T cells [10]. Exogenous expression of Claudin1 (CLDN1), microRNA-122 (miR-122), and exchangeable apolipoproteins is required for entry, viral RNA replication, and particles formation, respectively. In 293T cells stably expressing CLDN1 and miR-122 (293T/CLDN/miR-122), expression of E^rns^ facilitated the formation of infectious HCV particles to a similar extent as ApoE expression, but had no effect on the replication of viral RNA (Fig 1E–1G and S1 Fig). Next, to determine the effect of E^rns^ expression on the entry and replication process of HCV, infectivity of HCV pseudotype particles (HCVpp) and replication of sub-genomic replicon (SGR) RNA were examined in the BE-KO cells expressing HA-E^rns^. Expression of either HA-E^rns^ or ApoE in the BE-KO cells exhibited no effect on the entry of HCVpp (S3A Fig) or the colony formation of SGR RNA (S3B Fig). In addition, expression of E^rns^ had no effect on the propagation of HCV in parental Huh7 cells (S4 Fig). These results suggest that E^rns^ can compensate for the role of apolipoproteins in HCV propagation independent of entry and viral RNA replication.

### The C-terminal amphipathic α-helix of E^rns^ is crucial for the formation of infectious HCV particles

**Fig 2:**
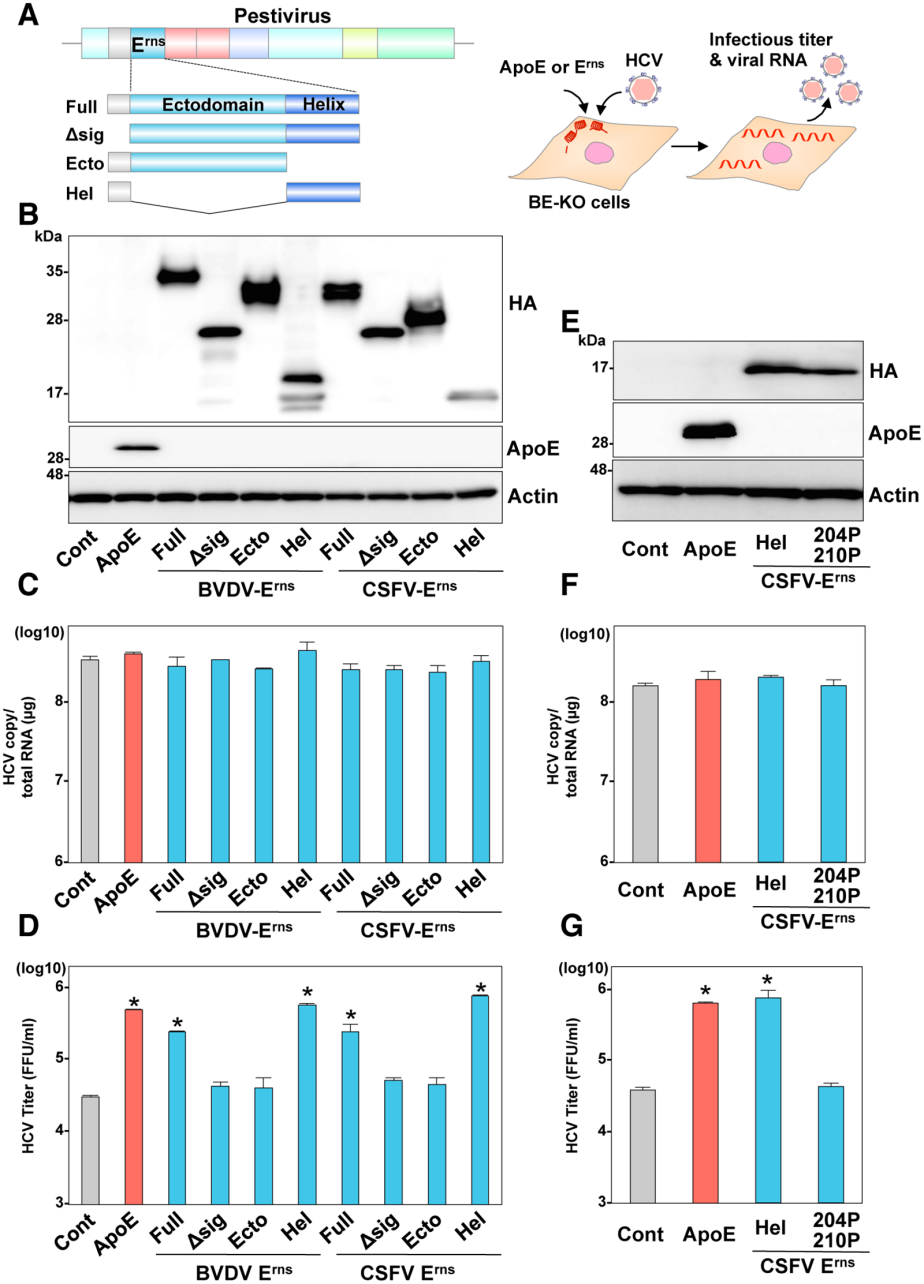
The C-terminal amphipathic α-helix in E^rns^ is important to compensation for the role of apolipoproteins in the infectious particle formation of HCV. (A) The cDNA constructs for expression of HA-tagged E^rns^ (HA-E^rns^) mutants; the full length of E^rns^ with the signal sequence of the C-terminal 30 amino acids of core protein (E^rns^), and without the signal sequence (Δsig), the ectodomain of E^rns^ with the signal sequence (Ecto), and the C-terminal amphipathic α-helix of E^rns^ (Hel). (B) Expression of ApoE and the HA-E^rns^ mutants was determined by immunoblotting at 48-h post-transduction of lentiviruses into the BE-KO cells. Intracellular HCV RNA (C) and extracellular infectious titers (D) were determined at 72-h post-infection with JFH1 HCV at an MOI of 1 by qRT-PCR and focus-forming assay, respectively. (E) The insertion of two proline residues (204P/210P) in the Hel mutant containing the C-terminal amphipathic α-helix of HA-E^rns^ of CSFV was generated to examine the significance of the membrane-binding ability in the formation of HCV particles. Expression of Hel, Hel (204P/210P), and ApoE was determined by immunoblotting at 48-h post-transduction of lentiviruses into the BE-KO cells. Intracellular HCV RNA (F) and extracellular infectious titers (G) were determined at 72-h post-infection with JFH1 HCV at an MOI of 1 by qRT-PCR and focus-forming assay, respectively. In all cases, asterisks indicate significant differences (* *p* < 0.01) versus the results of the control cells.

The C-terminal α-helix domain of E^rns^ is considered to participate in the formation of infectious particles by functioning as an anchor to viral particles [5, 12]. Thus, to examine whether the α-helices of E^rns^ can compensate for the roles of exchangeable apolipoproteins in formation of infectious HCV particles, the BE-KO cells expressing deletion mutants of E^rns^ derived from BVDV and CSFV were infected with HCV (Fig 2A). Expression of the HA-tagged either the full-length (Full) or truncated mutants lacking an ectodomain but carrying the C-terminal α-helices (Hel) of HA-E^rns^ recovered the production of infectious particles in the BE-KO cells, but expression of the HA-E^rns^ mutants carrying only an ectodomain (Ecto) or missing the signal sequence of the core protein (Δsig) did not recover the production, and none of the mutants affected the viral RNA replication at 72-h post-infection (Fig 2B–2D), suggesting that the amphipathic α-helices of E^rns^ can compensate for the roles of apolipoproteins in HCV particle formation. In agreement with the previous report indicating that insertion of proline residues at positions 204 and 210 in the C-terminus impaired the amphipathic α-helical characteristics of E^rns^ [13], transfection of an *in vitro*-transcribed mutant CSFV RNA with insertion of these proline residues in E^rns^ (204P/210P) did not result in production of infectious CSFV in the porcine kidney-derived cell line, SK6 (S5 Fig). Next, to examine the roles of amphipathic α-helices of E^rns^ in the infectious particle formation of HCV, the BE-KO cells expressing either the C-terminal α-helices (Hel) of CSFV HA-E^rns^ or those carrying the insertion of the proline residues (204P/210P) were infected with HCV, and intracellular HCV RNA and infectious titers in the supernatants were determined at 72-h post-infection (Fig 2E–2G). Infectious titers of HCV in the BE-KO cells expressing the 204P/210P mutant were significantly lower than those in the BE-KO cells expressing either the C-terminal α-helices of HA-E^rns^ or ApoE, while intracellular HCV RNA replication was comparable among these cells. These results strongly suggest that the amphipathic α-helices of E^rns^ are crucial in allowing E^rns^ to compensate for the role of apolipoproteins in the infectious particle formation of HCV in BE-KO cells.

### Apolipoproteins can partially compensate for the role of E^rns^ in the formation of infectious particles of pestivirus

**Fig 3:**
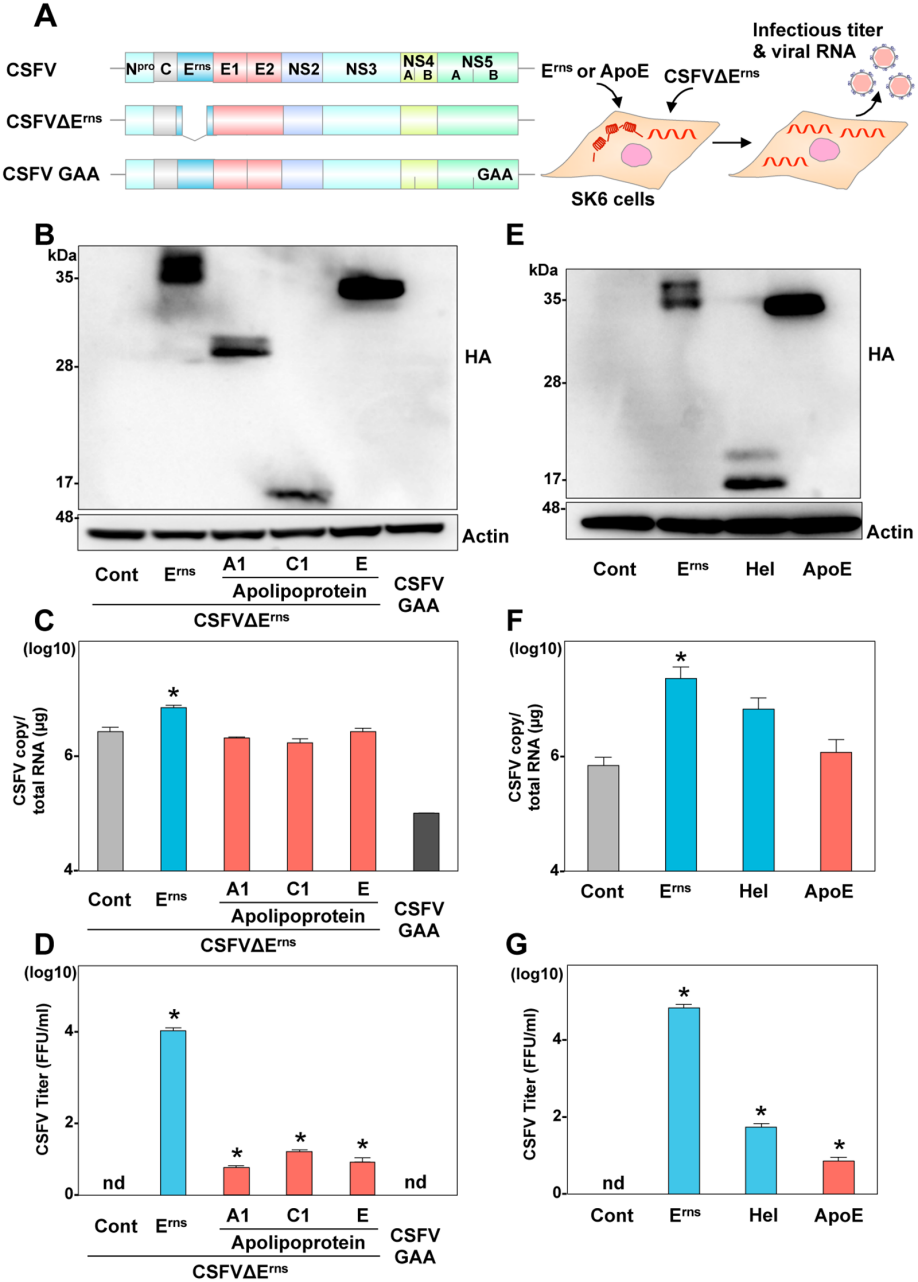
Exchangeable apolipoproteins can compensate for the role of E^rns^ in the infectious particle formation of pestivirus. (A) Schematics of the wild type, E^rns^ deletion (CSFVΔE^rns^), and polymerase dead (GAA) CSFV RNA, and the experimental procedure. (B) Expression of the full length of HA-tagged E^rns^ (HA-E^rns^), HA-ApoA1, HA-ApoC1, and HA-ApoE was determined by immunoblotting at 48-h post-transduction of lentiviruses into SK6 cells. Intracellular CSFV RNA (C) and extracellular infectious titers (D) were determined at 72-h post-electroporation with CSFVΔE^rns^ by qRT-PCR and focus-forming assay, respectively. (E) Expression of HA-E^rns^, Hel, and HA-ApoE was determined by immunoblotting at 48-h post-transduction of lentiviruses into SK6 cells. Intracellular CSFV RNA (F) and extracellular infectious titers (G) were determined at 72-h post-electroporation with CSFVΔE^rns^ by qRT-PCR and focus-forming assay, respectively. In all cases, asterisks indicate significant differences (* *p* < 0.01) versus the results of the control cells.

It has been well documented that trans-complementation of E^rns^ can function in propagation of CSFV carrying a deletion of E^rns^ in SK6 cells [14]. In the present study, therefore, we first confirmed that exogenous expression of secretory HA-E^rns^ rescued the formation of infectious particles upon electroporation of the E^rns^-deleted CSFV (CSFVΔE^rns^) RNA (Fig 3A) in SK6 cells (S6 Fig). To examine whether exchangeable apolipoproteins can compensate for the role of E^rns^ in the production of infectious pestiviral particles, the CSFVΔE^rns^ RNA was electroporated into SK6 cells expressing either exchangeable apolipoproteins or HA-E^rns^, and intracellular viral RNA and infectious titers in the supernatants were determined (Fig 3A–3D). Surprisingly, the HA-tagged apolipoproteins, ApoA1, ApoC1, and ApoE, could rescue infectious particle formation of the incapable mutant CSFVΔE^rns^. Next, to examine the role of amphipathic α-helices in the formation of pestivirus particles, either a full-length or a mutant lacking the ectodomain of HA-E^rns^ (Hel) or HA-ApoE was expressed in SK6 cells, and intracellular viral RNA and infectious titers in the supernatants were determined at 72-h post-electroporation with the CSFVΔE^rns^ RNA (Fig 3E–3G). Although infectious particle formation in the supernatants of cells expressing HA-tagged either the C-terminal α-helices of E^rns^ or ApoE was lower than that in the cells expressing the HA-tagged full-length E^rns^, substantial infectivity was recovered, suggesting that exchangeable apolipoproteins have a common role with the C-terminal amphipathic α-helices of E^rns^ in the infectious particle formation of pestiviruses.

### NS1 also has common function with apolipoproteins and E^rns^ in infectious viral particle formation

**Fig 4:**
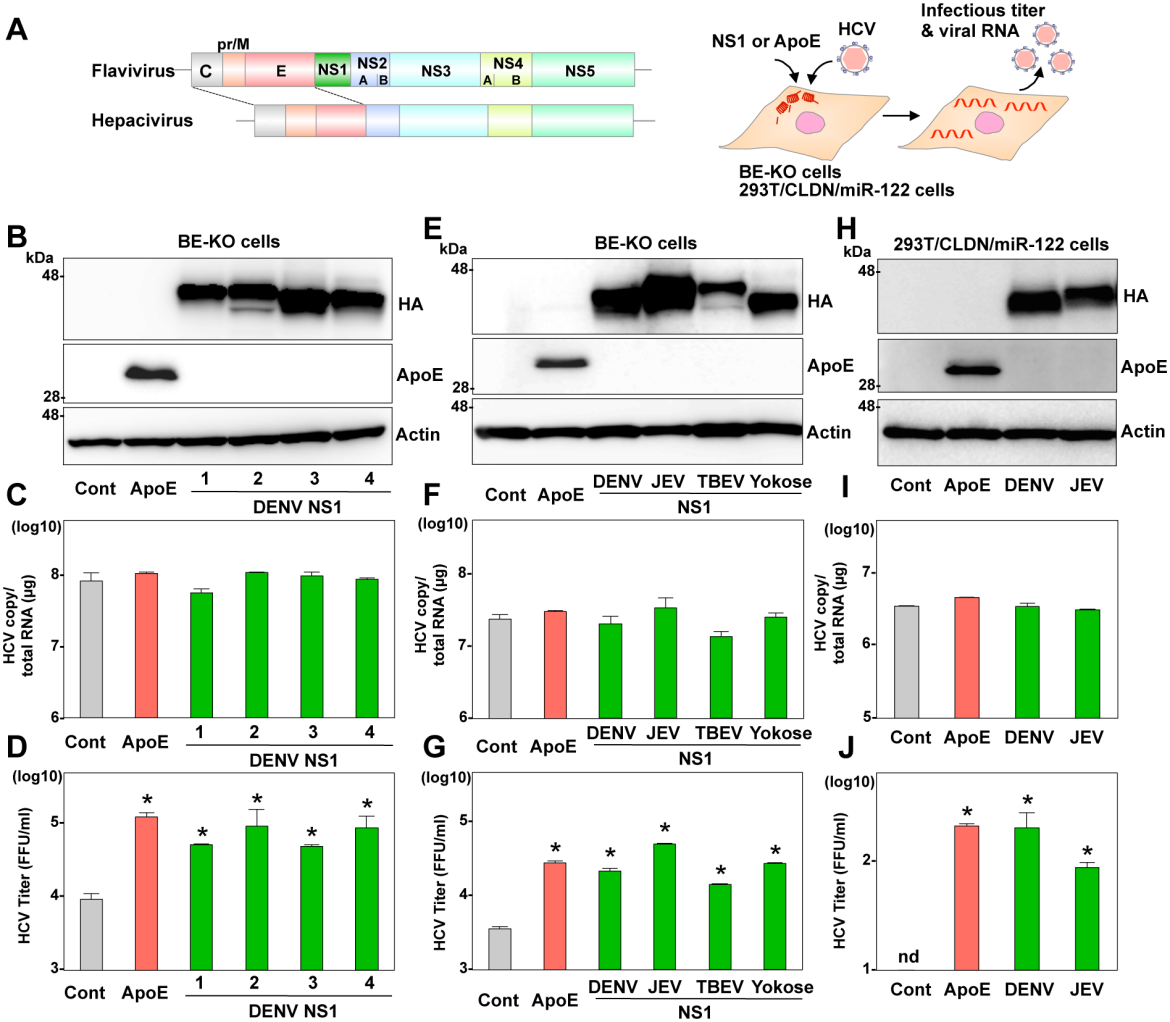
Flavivirus NS1 can compensate for the role of apolipoproteins in the infectious particle formation of HCV. (A) Gene structures of flavivirus and hepacivirus and the experimental procedure. (B) Expression of ApoE and HA-tagged NS1 (HA-NS1) from serotypes 1 to 4 of DENV was determined by immunoblotting at 48-h post-transduction of lentiviruses into the BE-KO cells. Intracellular HCV RNA (C) and extracellular infectious titers (D) were determined at 72-h post-infection with JFH1 HCV at an MOI of 1 by qRT-PCR and focus-forming assay, respectively. (E) Expression of ApoE and HA-NS1 from DENV, JEV, TBEV, and Yokose virus was determined by immunoblotting at 48-h post-transduction of lentiviruses into the BE-KO cells. Intracellular HCV RNA (F) and extracellular infectious titers (G) were determined at 72-h post-infection with JFH1 HCV at an MOI of 1 by qRT-PCR and focus-forming assay, respectively. (H) Expression of ApoE and HA-NS1 from DENV and JEV in 293T/CLDN1/miR-122 cells was determined by immunoblotting analysis. Cells were infected with HCV at an MOI of 10, and intracellular HCV RNA (I) and infectious titers in the supernatants (J) at 72-h post-infection were determined by qRT-PCR and focus-forming assay, respectively. In all cases, asterisks indicate significant differences (* *p* < 0.01) versus the results of the control cells.

Recently, it was shown that the secretory glycoprotein of flavivirus NS1 has dual functions in viral replication and infectious particle formation [7, 8]. Therefore, we considered that NS1 might also be capable of compensating for the function of exchangeable apolipoproteins and E^rns^ in the particle formation of hepaciviruses and pestiviruses, respectively. Exogenous expression of the HA-tagged NS1 (HA-NS1) derived from DENV serotypes 1 to 4 rescued the infectious particle formation of HCV in the culture supernatants of the BE-KO cells at 72-h post-infection with HCV, while viral RNA replication was comparable among these cells (Fig 4A–4D). To analyze the role of NS1 in more detail, effects of exogenous expression of HA-NS1 derived from DENV, JEV, TBEV, and Yokose virus in the BE-KO cells on the infectious particle formation of HCV were determined. The formation of infectious particles of JFH1 virus (Fig 4E–4G) as well as Con1/JFH1 chimeric virus (S7 Fig) in the BE-KO cells was enhanced by the expression of HA-NS1 derived from these viruses, while the efficiencies of viral RNA replication were comparable. As seen in E^rns^ expression, exogenous expression of HA-NS1 derived from DENV and JEV also facilitated the formation of infectious HCV particles in the 293T/CLDN-miR122 cells, while intracellular HCV RNA levels were not affected by the expression of HA-NS1 (Fig 4H–4J). In addition, expression of HA-NS1 exhibited no effect on the entry of HCVpp (S8A Fig) or replication of SGR RNA (S8B Fig). These results suggest that NS1 also possesses functions similar to those of exchangeable apolipoproteins in the particle formation of HCV.

**Fig 5:**
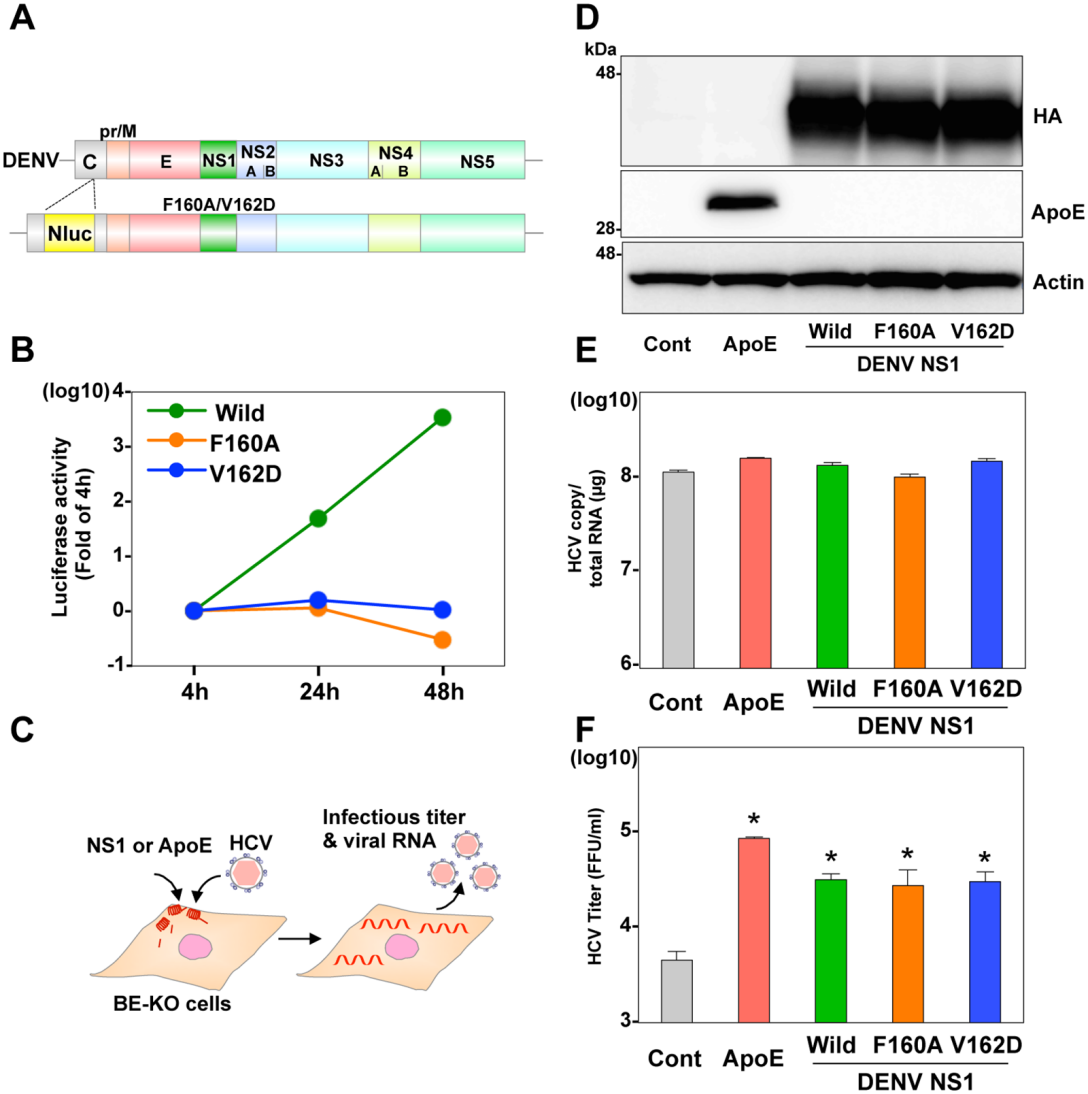
NS1 mutants in the hydrophobic protrusion deficient in viral replication can compensate for the role of ApoE in the infectious particle formation of HCV. (A) Gene structure of a recombinant DENV with a luciferase gene and possessing a single amino-acid substitution in NS1 (F160A or V162D). (B) Luciferase activity was determined at 4, 24, and 48-h post-electroporation with the recombinant DENV RNA in BE-KO cells. (C) Experimental procedure. (D) Expression of ApoE and the wild type and mutant HA-tagged NS1 was determined by immunoblotting at 48-h post-transduction of lentiviruses into the BE-KO cells. Intracellular HCV RNA (E) and extracellular infectious titers (F) were determined at 72-h post-infection with JFH1 HCV at an MOI of 1 by qRT-PCR and focus-forming assay, respectively. In all cases, asterisks indicate significant differences (* *p* < 0.01) versus the results of the control cells.

Previous reports have shown that mutations in the hydrophobic protrusion (F160A or V162D) of NS1 impaired viral RNA replication, but had no effect on the remodeling of liposome by incubation with recombinant NS1 [15]. To examine this issue, we first attempted to confirm that introduction of the mutations in the NS1 completely abolished the replication of DENV RNA, in contrast to the efficient replication of the wild type RNA (Fig 5A and 5B). To examine the role of membrane-binding ability of NS1 in the particle formation of HCV, infectious titers in the supernatants of the BE-KO cells exogenously expressing these HA-NS1 mutants were determined upon infection with HCV (Fig 5C–5F). The expression of HA-NS1 irrespective of the introduction of mutations rescued the formation of infectious HCV particles in the BE-KO cells, suggesting that NS1 can compensate for the roles of apolipoproteins in the formation of infectious HCV particles by different mechanisms of replication complex formation.

**Fig 6:**
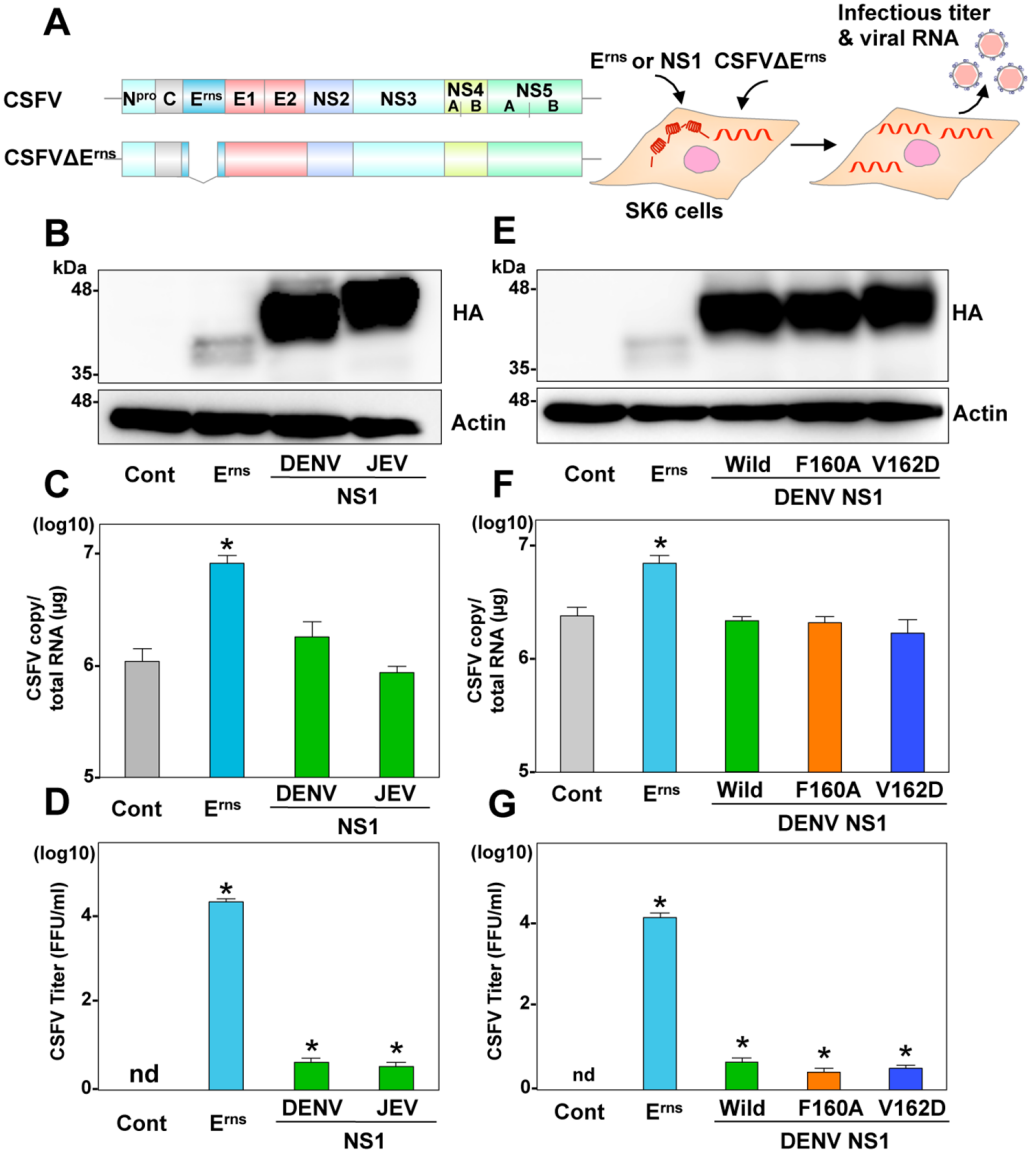
NS1 can compensate for the role of E^rns^ in the infectious particle formation of CSFV. (A) Schematics of the wild type and E^rns^ deletion (CSFVΔE^rns^) CSFV RNA, and the experimental procedure. (B) Expression of HA-tagged E^rns^ (HA-E^rns^) and HA-NS1 from DENV and JEV was determined by immunoblotting at 48-h post-transduction of lentiviruses into SK6 cells. Intracellular CSFV RNA (C) and extracellular infectious titers (D) were determined at 72-h post-electroporation with CSFVΔE^rns^ by qRT-PCR and focus-forming assay, respectively. (E) Expression of HA-E^rns^, and the wild type and mutant (F160A and V162D) HA-NS1 from DENV was determined by immunoblotting. Intracellular CSFV RNA (F) and extracellular infectious titers (G) were determined at 72-h post-electroporation with CSFVΔE^rns^. In all cases, asterisks indicate significant differences (* *p* < 0.01) versus the results of the control cells.

Next, to examine the role of NS1 in the particle formation of pestivirus, NS1 from DENV and JEV was expressed in SK6 cells electroporated with the CSFVΔE^rms^ RNA and intracellular viral RNA and infectious particle formations in the supernatants were determined (Fig 6A–6D). The HA-NS1 of DENV and JEV rescued the formation of infectious particles, but quite slightly less than 0.01% of rescue efficiency compared with the homologous E^rns^. These expressions exhibited no effect on viral RNA replication. In addition, expression of the HA-NS1 mutants (F160A or V162D) of DENV in cells transduced with the CSFVΔE^rns^ RNA also partially rescued the particle formation of CSFV (Fig 6E–6G), suggesting that NS1 can substantially but not completely function as the role of E^rns^ in the infectious particle formation of pestiviruses. Taken together, these data suggest that NS1 participates in the infectious particle formation independent from the replication complex formation.

**Fig 7:**
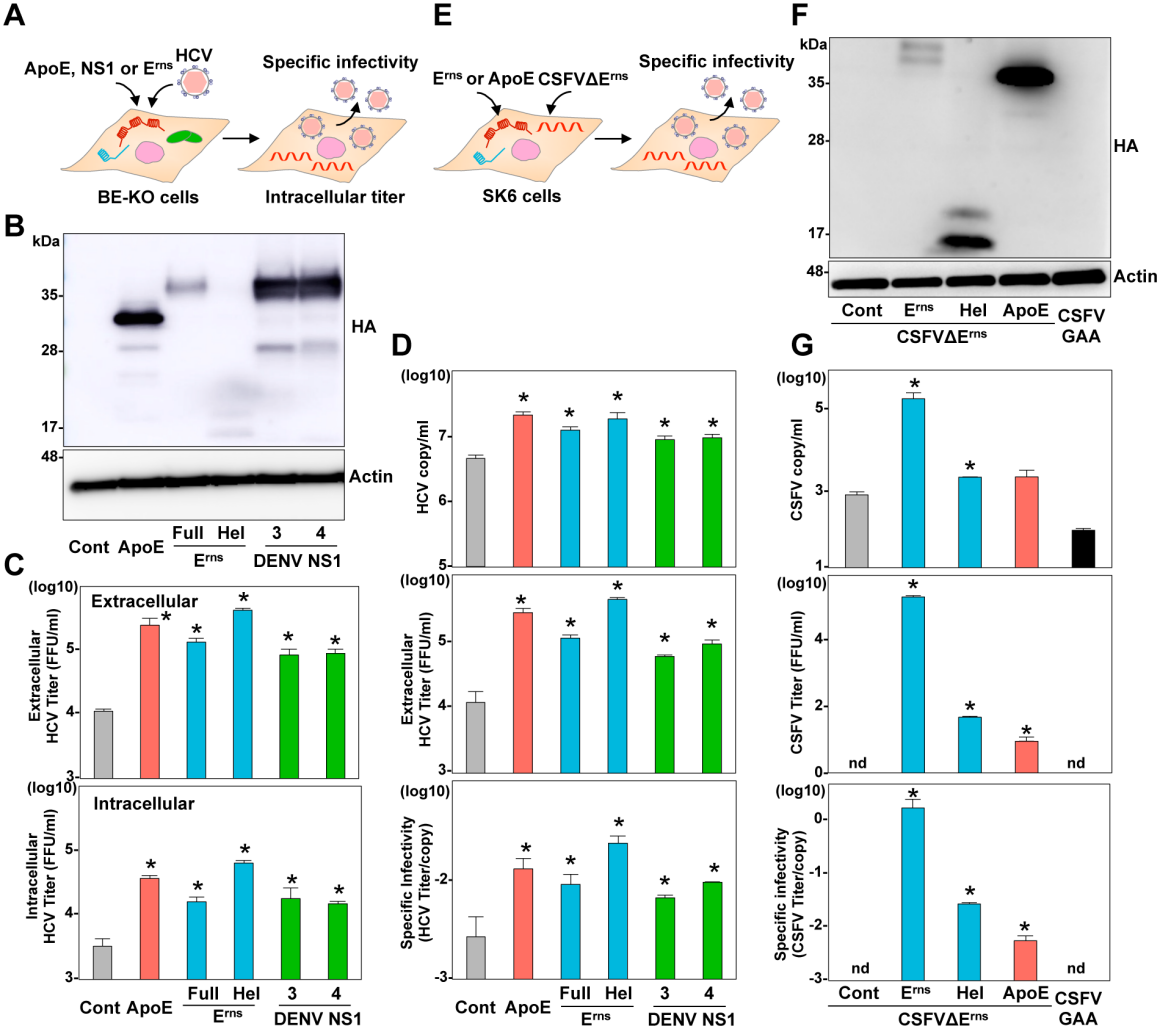
ApoE, E^rns^, and NS1 participate in the infectious particle formation. (A) Experimental procedure. (B) Expression of HA-tagged ApoE (HA-ApoE), the full length of HA-E^rns^, the C-terminal amphipathic α-helix of HA-E^rns^ (Hel), and HA-NS1 from serotype 3 and 4 of DENV was determined by immunoblotting at 48-h post-transduction of lentiviruses into BE-KO cells. (C) Intracellular and extracellular infectious titers were determined at an MOI of 1 by a focus-forming assay. (D) Specific infectivity was calculated as extracellular infectious titers/extracellular HCV RNA copies in BE-KO cells expressing HA-ApoE, HA-E^rns^, Hel, and HA-NS1 at 72-h post-infection. (E) Experimental procedure. (F) Expression of the HA-tagged E^rns^, Hel, and ApoE was determined by immunoblotting at 48-h post-transduction of lentiviruses into SK6 cells. (G) Extracellular CSFV RNA and infectious titers were determined at 72-h post-electroporation with CSFVΔE^rns^ RNA by qRT-PCR and focus-forming assay, respectively. Specific infectivity was calculated as extracellular infectious titers/extracellular CSFV RNA copies in SK6 cells. In all cases, asterisks indicate significant differences (* *p* < 0.01) versus the results of the control cells.

So far, we found that the secretory glycoproteins, ApoE, E^rns^ and NS1, commonly participate in the formation of infectious HCV or pestivirus particles. To examine further detail of the roles of these secretory glycoproteins at the late stage of viral life cycle, intracellular infectivity and specific infectivity (infectious titers/viral RNA levels) in the supernatant were determined in BE-KO cells expressing HA-ApoE, HA-E^rns^, and Hel derived from CSFV, or HA-NS1 derived from DENV serotypes 3 and 4 at 72-h post-infection with HCV (Fig 7A and 7B). Both intracellular and extracellular infectious titers were enhanced by the expression of the proteins, suggesting that these viral glycoproteins participate in the intracellular particle formation (Fig 7C). In addition, specific infectivity of HCV was also enhanced by the expression of the viral proteins, indicating that these secretory glycoproteins participate in the maturation of infectious HCV particles (Fig 7D). Next to investigate the roles of these secretory glycoproteins in the maturation of pestivirus particles, CSFVΔE^rns^ RNA was electroplated into the SK6 cells expressing either HA-E^rns^, HA-Hel, or HA-ApoE, and specific infectivity in the supernatants was determined at 72-h post electroporation (Fig 7E and 7F). Specific infectivity of the non-infectious CSFVΔE^rns^ RNA was rescued by the exogenous expression of either E^rns^ or ApoE, suggesting that the heterogenous secretory glycoproteins can partially but substantially compensate roles of E^rns^ in maturation of the infectious particles of CSFV (Fig 7G).

**Fig 8:**
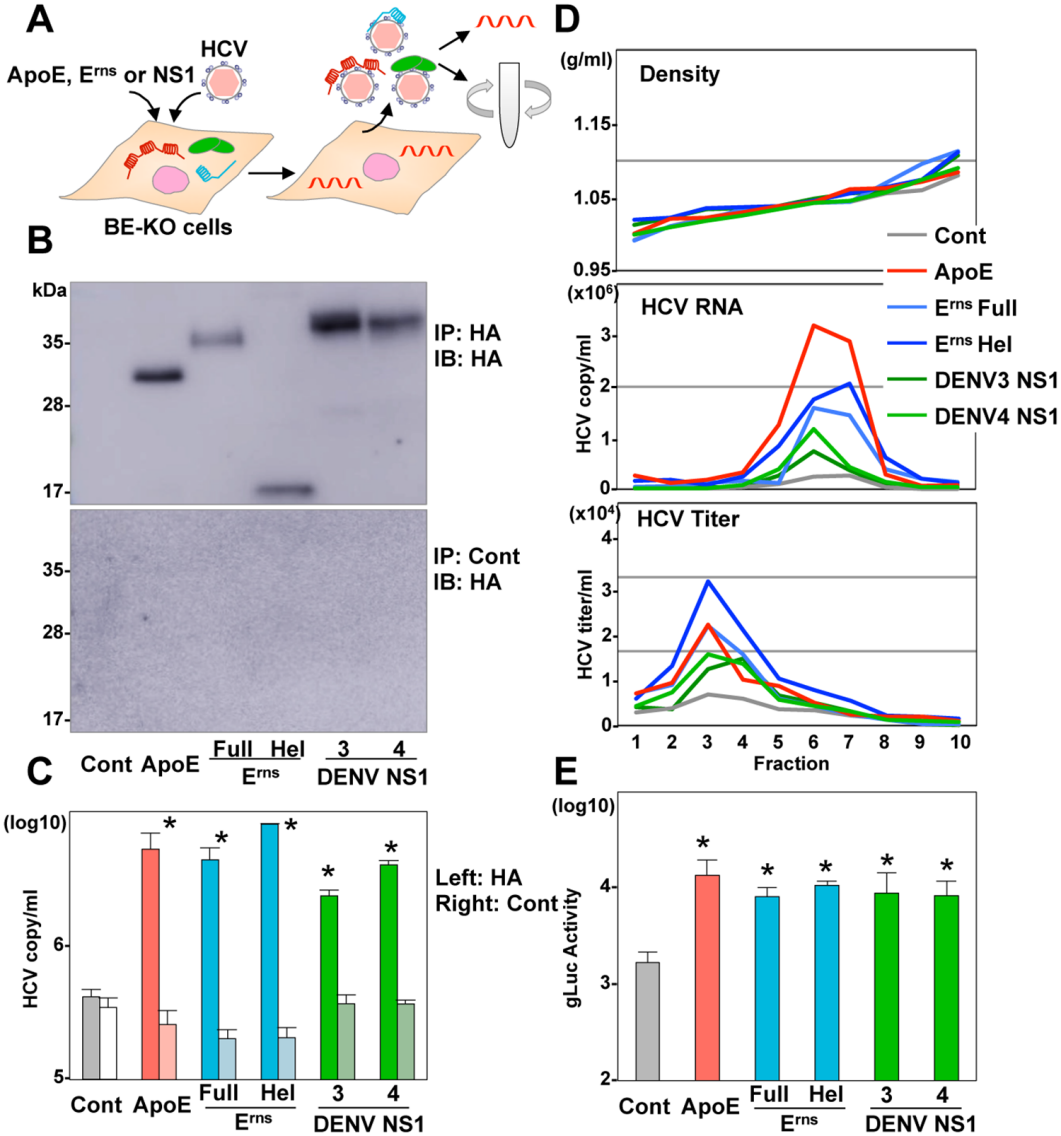
Characterization of HCV particles produced in BE-KO cells expressing either ApoE, E^rns^ or NS1. (A) Experimental procedure. (B) HA-tagged complexes were immunoprecipitated from the supernatants of BE-KO cells expressing either HA-ApoE, HA-E^rns^, Hel, or HA-NS1 by using a control anti-IgG or anti-HA antibody. Expression of HA-ApoE, HA-E^rns^, Hel, or HA-NS1 in the immunoprecipitated samples was determined by immunoblotting. (C) The HCV RNA associated with ApoE, E^rns^, and NS1 was determined by qRT-PCR. (D) The supernatants of BE-KO cells expressing either HA-ApoE, HA-E^rns^, Hel, or HA-NS1 upon infection with HCV at an MOI of 1 were subjected to density gradient fractionation, and density (upper), HCV RNA levels (middle) and infectious titers (bottom) for each fraction were determined by qRT-PCR and focus forming assay, respectively. (E) Transcomplemented HCV particles (HCVtcp) were produced in BE-KO cells expressing either HA-ApoE, HA-E^rns^, HA-Hel or HA-NS1 at 72-h post-transfection with pCAGC-NS2/JFH1am and PHH SGR-JFH1/Gluc/NS3m. HCVtcp were inoculated into Huh7.5.1 cells, and gaussia luciferase (gLuc) activities were determined at 72-h post-infection. In all cases, asterisks indicate significant differences (* *p* < 0.01) versus the results of the control cells.

Finally, to characterize the viral particles produced in BE-KO cells expressing either ApoE, E^rns^, or NS1, association of the glycoproteins with viral particles was determined by immunoprecipitation and qRT-PCR. Large amounts of HCV RNA were recovered in the immunoprecipitates with an anti-HA antibody but not with a control antibody in BE-KO cells expressing HA-ApoE, HA-E^rns^, and HA-NS1, suggesting that not only ApoE but also E^rns^ and NS1 directly bind to HCV particles (Fig 8A–8C). In addition, buoyant density gradient analysis revealed a similar distribution of viral RNA and infectious titers in the supernatants of BE-KO cells expressing either ApoE, E^rns^, Hel, or NS1 (Fig 8D). Furthermore, there was no significant difference in the physical properties of the pestivirus particles produced in SK6 cells expressing E^rns^ and ApoE (S9 Fig). Next, to examine the role of the interaction of the glycoproteins with viral particles on the entry process, infectivity of trans-complemented HCV particles (HCVtcp) obtained from BE-KO cells expressing either ApoE, E^rns^, or NS1 was compared. Interestingly, infectivity of HCVtcp generated in BE-KO cells expressing either E^rns^ or NS1 was comparable to that in those expressing ApoE (Fig 8E). Collectively, these results suggest that E^rns^ and NS1 play similar roles with ApoE in the particle formation of HCV and CSFV.

## Discussion

In this study, we investigated the common roles of virus- and host-derived secretory membrane-binding proteins in the viral particle formation of viruses of the family *Flaviviridae* (S10 Fig). The viral secretory glycoproteins E^rns^ and NS1 play important roles in the formation of infectious particles of pestiviruses and flaviviruses, respectively. In contrast, HCV utilizes host factors including exchangeable apolipoproteins [10]. The present data suggest that viral proteins were converged to host factors in the evolutional process of *Flaviviridae* viruses, and utilization of host factors in the viral life cycle is closely associated with the tissue- and species-specificities among *Flaviviridae* viruses.

E^rns^ and NS1 are unique in pestiviruses and flaviviruses, respectively, and are enigmatic in *Flaviviridae* viruses in general, since their function has not been fully elucidated compared to other viral proteins such as core, envelope, protease, and polymerase [5]. E^rns^ contains an amphipathic helix domain of 61 residues for membrane binding instead of a transmembrane [12]. Although the current study revealed that the C-terminal helix domain of E^rns^ plays a crucial role in the infectious particle formation, the helix domains from exchangeable apolipoproteins and CAMP have been shown to play similar roles in particle formation [10, 11]. The effects of exogenous expression of the C-terminal α-helices of E^rns^ on HCV and CSFV particle formation were comparable to those of ApoE; however, the absence of an ectodomain of E^rns^ suppressed the efficiency of infectious particle formation of the pestivirus (Fig 3G), suggesting that the amphipathic helices of E^rns^ possess a function in common with apolipoproteins but the ectodomain plays a different role in the life cycle of pestivirus. Initially, NS1 was considered to participate in assembly and release of flavivirus particles due to its localization in the ER lumen and secretion profile [16]. However, the introduction of reverse genetics into flavivirus research revealed that NS1 plays a crucial role in formation of the replication complex through its interaction with NS4A and NS4B in the ER lumen [17, 18]. A recent study on DENV showed that several mutations in NS1 impaired the production of infectious particles but had no effect on viral RNA replication [7]. In addition, transposon mutagenesis of the Zika virus genome exhibited the requirement of NS1 at the late stage of viral life cycle [8]. On the other hand, Akey *et al*. showed that NS1 mutants that are unable to generate replication complexes are nonetheless capable of remodeling the membranes of liposomes [15]. In this study, we showed that NS1 participates in the particle formation of HCV and CSFV, but not in the replication of viral RNA. Collectively, these data suggest that NS1 plays dual roles in the formation of replication complex and infectious particles through different mechanisms. Secretory glycoproteins were interacted with HCV particle and enhanced the infectious particle production, suggesting that viral NS1, E^rns^ and host-derived secretory glycoproteins commonly have a function in, at least, virus particle maturation leading to support release and stability of the virus.

Flaviviruses and pestiviruses have a broad tissue range of infection, while hepaciviruses exhibit strong hepatic tropism [2–4]. Several host factors other than exchangeable apolipoproteins are also involved in the liver tropism of HCV infection [4, 19]. CLDN1 and miR-122, which participate in entry and replication of the viral genome, respectively, are mainly expressed in the liver. Considering the abundant expression of the exchangeable apolipoproteins in the liver, HCV might have evolved to utilize the apolipoproteins in the formation of infectious particles through deletion of the viral genome that encodes secretory glycoproteins, leading to acquire liver tropism as well as to utilize miR-122 and CLDN1. In contrast to hepaciviruses, pegiviruses exhibit PBMC tropism [20]. Recently, we reported that CAMP, which is highly expressed in bone marrow and in many cell types related to the immune system, plays a role similar to ApoE in the formation of infectious HCV particles [11]. It is feasible that an abundant expression of CAMP participates in the tropism of pegivirus infection and the lack of secretory viral proteins. In addition, pegiviruses have lost almost all their capsid genes in the course of evolution, suggesting that other host or exogenous factors in PBMC compensate for the role of the core protein in the formation of pegivirus particles [21]. Recently, Zhang *et al*. reported that a capsidless single-stranded RNA virus hijacks capsid proteins from another double-stranded RNA virus for its propagation [22]. Further studies are required to clarify whether endogenous or exogenous factors were utilized for the formation of pegivirus particles.

Previous reports have shown that amphipathic helices of exchangeable apolipoproteins play important roles in the formation of infectious HCV particles through their interaction with the viral membrane, and the association of the apolipoproteins with HCV particles has been detected in the sera of hepatitis C patients and culture supernatants [10, 23, 24]. The present study clearly indicated that the enhancement of infectious particle formation by the expression of apolipoproteins has similarity to the enhancement of infectious particle formation by E^rns^. In addition, infectivity of HCVtcp produced in BE-KO cells expressing either E^rns^ or NS1 was comparable with that in those expressing ApoE. Interestingly, both ApoE and E^rns^ bind to viral particles through their interaction with heparan sulfate, which is an entry factor for HCV and CSFV [25, 26]. DENV has also been shown to bind to the cell surface through the interaction of NS1 with heparan sulfate and to utilize laminin receptor for an efficient entry, as seen in the entry of pestiviruses through the interaction of E^rns^ and the receptor [27–29]. Considering the interaction of these glycoproteins with surface proteins of plasma membrane, NS1 and E^rns^ play a similar role with apolipoproteins in the entry of HCV. Recent reports showed that ApoE associated with viral particles enhances infectivity of HCV [30, 31], therefore, non-cell derived exchangeable apolipoproteins in the culture medium might participate in the enhancement of infectivity of HCV through the interaction with viral particles. In contrast, expression of ApoE and NS1 has shown to compensate the particle formation of pestivirus *in trans*, however, the efficiency of recovery was significantly low compared with that of E^rns^. The amphipathic helices of E^rns^ retain a common function in particle formation with apolipoproteins but E^rns^ might be evolved to optimize the structural element for an efficient replication of pestivirus.

In conclusion, we have shown that the membrane-binding glycoproteins, including NS1, E^rns^, and exchangeable apolipoproteins, play common roles in the life cycle of viruses in *Flaviviridae*. This may help to reveal new mechanisms in the tissue tropism and evolution of *Flaviviridae* viruses. However, further studies are needed to elucidate the biological significance of the enigmatic viral proteins E^rns^ and NS1 in the viral life cycle.

## Materials and methods

### Plasmids

The cDNA clones of ApoE, pestiviral E^rns^ from BVDV, CSFV, and BDV, flaviviral NS1 from JEV, DENV, TBEV, and Yokose virus, and AcGFP were inserted between the *Xho*I and *Xba*I sites of the lentiviral vector pCSII-EF-RfA by using Infusion technique, and the resulting plasmids were designated as pCSII-EF-ApoE, pCSII-EF-E^rns^, pCSII-EF-NS1, and pCSII-EF-GFP, respectively. The mutant cDNAs of E^rns^ and NS1 were amplified by PCR and inserted into pCSII-EF. The plasmid pHH-JFH1 encodes a full-length cDNA of the JFH1 strain (GenBank accession number: AB047639) [32]. pHH-JFH1-E2p7NS2mt contains three adaptive mutations in pHH-JFH1, and pSGR-JFH1 encodes subgenomic cDNA of the JFH1 strain. The CSFV was derived from the full-length cDNA clone of the GPE^−^ strain (GenBank accession number: D49533) [33]. The plasmids encoding CSFVΔE^rns^, the 204P and 210P mutants of E^rns^ and the polymerase dead (GAA) mutant of CSFV were constructed by using a KOD-Plus-Mutagenesis Kit (Toyobo) and the respective oligonucleotide primers. The plasmid pMW119-DV4 Nluc sec encodes a full-length infectious clone of the DENV serotype 4 H241 strain (GenBank accession number: AY947539) containing a NanoLuc-luciferase gene in-frame insertion into the viral C gene as described previously [34]. The cDNA clones encoding the viral sequence for transfection were flanked by a modified T7 promoter sequence at the 5′ end and *Not*I restriction site at the 3′ end. The plasmid pX330 encoding hCas9 and sgRNA was obtained from Addgene (Addgene plasmid 42230). The fragments of guided RNA targeting the ApoB and ApoE gene were inserted into the *Bbs1* site of pX330 and designated pX330-ApoB and pX330-ApoE, respectively. The plasmids used in this study were confirmed by sequencing with an ABI 3130 genetic analyser (Life Technologies).

### Cell lines

All cell lines were cultured at 37°C under the conditions of a humidified atmosphere and 5% CO_2_. The human hepatocellular carcinoma-derived Huh7, human embryonic kidney-derived 293T cells, and African green monkey kidney-derived VeroE6 cells were maintained in DMEM (Nakarai) supplemented with 100 U/ml penicillin, 100 μg/ml streptomycin, and 10% fetal bovine serum (FBS). Huh7 cells were provided by the RIKEN BRC through the National Bio-Resource Project of MEXT, Japan. 293T and Vero cells were obtained from the American Type Culture Collection. The Huh7-derived cell line Huh7.5.1 was kindly provided by F. Chisari. The porcine kidney-derived SK6 cells [35] were propagated in DMEM supplemented with 100U/ml penicillin, 100 μg/ml streptomycin, 5% BVDV antibody free FBS (Japan Bio Serum) and 5% horse serum (Life Technologies). Huh7 cells were transfected with pX330-ApoE and pX330-ApoB by Trans IT LT-1 (Mirus), and single cell clones were established. To screen for gene-knockout Huh7 cell clones, mutations in the target loci were determined by using a Surveyor assay (Transgenomic) according to the manufacturer’s protocol. Frameshift of the genes and deficiencies of protein expressions were confirmed by direct sequencing and immunoblotting analysis, respectively.

### Antibodies

Mouse monoclonal antibodies to β-actin, dsRNA, and ApoE were purchased from Sigma, English & Scientific Consulting Kft, and Santa Cruz, respectively. Rabbit anti-CLDN1 and Alexa Flour (AF) 488-conjugated anti-rabbit IgG antibodies were purchased from Life Technologies. Rat anti-HA antibody was purchased from Roche Diagnostics. Rabbit anti-HCV NS5A antibody and mouse monoclonal antibody 46/1 against pestiviral NS3 were generated as described previously [36, 37].

### *In vitro* transcription, RNA transfection, and colony formation

The plasmid pSGR-JFH1 digested with *Xba*I and blunted with mung bean exonuclease was transcribed *in vitro* by using a MEGAscript T7 kit (Life Technologies) according to the manufacturer's protocol. Capped and polyadenylated firefly luciferase (Fluc) RNAs were synthesized by using an mMESSAGE mMACHINE T7 Ultra kit (Life Technologies). The *in vitro* transcribed RNA (5 μg) was electroporated into cells at 5×10^6^ cells/0.4 ml under conditions of 190 V and 950 μF using a Gene Pulser (BioRad) and plated on DMEM containing 10% FBS. The medium was replaced with fresh DMEM containing 10% FBS and 1 mg/ml G418 at 24-h post-transfection of transcribed RNA. The remaining colonies were fixed with 4% paraformaldehyde and stained with crystal violet at 1-month post-electroporation.

### Preparation of viruses

pHH-JFH1-E2p7NS2mt was introduced into Huh7.5.1 cells, HCVcc in the supernatant was collected after serial passages, and infectious titers were determined by a focus-forming assay and expressed in focus-forming units (FFU). All of the cDNA-derived CSFV viruses were rescued as described previously [33]. The supernatants were collected from the electroplated cells and infectious titers were determined by a focus-forming assay. The infectious DENV clones linearized with *Not*I were transcribed by using an mMESSAGE mMACHINE T7 Ultra Kit, and the *in vitro* transcribed RNA was electroporated as described above. The supernatants were collected from the electroplated cells and infectious titers were determined by a focus-forming assay. HCVpp bearing E1 and E2 glycoproteins of JFH1 were produced as described previously [38]. HCVtcp were generated as described previously [39].

### Immunoblotting

Cells lysed on ice in lysis buffer (20 mM Tris-HCl [pH7.4], 135 mM NaCl, 1% Triton-X 100, 10% glycerol) supplemented with a protease inhibitor mix (Roche) were boiled in loading buffer and subjected to 5–20% gradient SDS-PAGE. The proteins were transferred to polyvinylidene difluoride membranes (Millipore) and incubated with the appropriate antibodies. The immune complexes were visualized with SuperSignal West Femto substrate (Thermo Scientific) and detected by use of an LAS-4000 image analyser (Fujifilm).

### Quantitative RT-PCR

For quantification of viral RNA copies, total RNA was extracted from cells by using a PureLink RNA Mini Kit (Life Technologies), and then first-strand cDNA synthesis and qRT-PCR were performed by using a TaqMan RNA-to-C_T_ 1-step Kit and ViiA7 system (Life Technologies), respectively, according to the manufacturer’s protocols. HCV RNA was quantified by using primers for TaqMan PCR targeted to the noncoding region of HCV as described previously [36]. For quantification of CSFV RNA, the primer set reported by Hoffmann *et al*. was employed [40]. Fluorescent signals were determined by the ViiA7 system.

### Immunoprecipitation

Culture supernatants of viral infected cells were incubated with anti-HA antibody beads or control IgG beads at 4°C for 2 h. The Sepharose beads were washed three times in PBS and RNAs were extracted using the Qiazol reagent (Qiagen). HCV-RNA associated with HA-tagged protein was detected by qRT-PCR as described as above.

### Buoyant density fractionation

Culture supernatants of viral infected cell were concentrated by using Spin-X UF concentrators (Corning), applied to the top of a linear gradient formed from 10 to 40% Optiprep (Axis-Shield) in PBS, and spun at 36,000 rpm for 16-h at 4°C by using an SW41 rotor (Beckman Coulter). Aliquots of 10 or 12 consecutive fractions were collected from top to bottom, and the density, infectious titer, and viral RNA level were determined for each fraction.

### Statistical analysis

The data for statistical analyses are the averages of two independent experiments. Results were expressed as the means ±standard deviations or standard errors. The significance of differences in the means was determined by Student's *t*-test.

## Supporting information

S1 Fig ApoE plays an important role in the infectious particle formation of HCV. (A) Experimental procedure. (B) Expression of CLDN1 and ApoE was determined by immunoblotting at 48-h post-transduction of lentiviruses into BE-KO and 293T cells. Intracellular HCV RNA (C) and extracellular infectious titers (D) were determined at 72-h post-infection with JFH1 HCV at an MOI of 1 (BE-KO cells) and 10 (293T cells) by qRT-PCR and focus-forming assay, respectively. In all cases, asterisks indicate significant differences (* *p* < 0.01) versus the results of the control cells.
(TIF)

S2 Fig Pestivirus E^rns^ can compensate for the role of apolipoproteins in the formation of infectious particles of Con1/JFH1 chimeric HCV. (A) Experimental procedure. Intracellular HCV RNA (B) and extracellular infectious titers (C) in BE-KO cells at 48-h post-transduction with lentiviruses were determined at 72-h post-infection with Con1/JFH1 chimeric HCV at an MOI of 1 by qRT-PCR and focus-forming assay, respectively. In all cases, asterisks indicate significant differences (* *p* < 0.01) versus the results of the control cells.
(TIF)

S3 Fig E^rns^ expression exhibits no effect on viral entry and replication of HCV RNA. (A) BE-KO cells expressing either ApoE or HA-tagged E^rns^ (HA-E^rns^) were inoculated with pseudotype particles bearing HCV envelope proteins E1 and E2 (HCVpp) (left) or VSV-G protein (VSVpp) (right), and luciferase activity was determined at 48-h post-infection. (B) Subgenomic HCV replicon RNA of the JFH1 strain was electroporated into the BE-KO cells with/without expression of ApoE or HA-E^rns^ by the lentiviral vectors, and the remaining colonies were fixed with 4% paraformaldehyde and stained with crystal violet at 1-month post-electroporation after selection with 1 mg/ml of G418.
(TIF)

S4 Fig Overexpression of ApoE, NS1 or E^rns^ in Huh7 cells exhibits no effect on RNA replication and infectious particles formation of HCV. (A) Experimental procedure. (B) Expression of ApoE, HA-tagged NS1 (HA-NS1) from JEV and DENV, and HA-E^rns^ from BVDV was determined by immunoblotting at 48-h post-transduction of lentiviruses into the BE-KO cells. Intracellular HCV RNA (C) and extracellular infectious titers (D) were determined at 72-h post-infection with JFH1 HCV at an MOI of 1 by qRT-PCR and focus-forming assay, respectively.
(TIF)

S5 Fig Proline insertion in the C-terminal helix of E^rns^ abolishes the production of infectious CSFV particles. (A) Schematics of CSFV of the wild type and mutants possessing insertion of proline (204P/210P) in E^rns^ and replacement in polymerase dead (GAA), and the experimental procedure. (B) Intracellular CSFV RNA and extracellular infectious titers (C) were determined at 72-h post-electroporation with CSFV RNA by qRT-PCR and focus-forming assay, respectively.
(TIF)

S6 Fig Expression of E^rns^ facilitates particle formation of CSFV in cells transfected with CSFVΔE^rns^ RNA. (A) Schematics of the wild type and E^rns^ deletion (CSFVΔE^rns^) RNA, and the experimental procedure. (B) Expression of HA-tagged E^rns^ (HA-E^rns^) with or without the signal sequence of the core protein (E^rns^ or Δsig E^rns^) was determined by immunoblotting at 48-h post-transduction of lentiviruses into SK6 cells. (C) Extracellular infectious titer in cells expressing either HA-E^rns^ or HA-Δsig E^rns^ was determined at 72-h post-electroporation with CSFVΔE^rns^ by focus-forming assay.
(TIF)

S7 Fig Flavivirus NS1 can compensate for the role of apolipoproteins in the formation of infectious particles of Con1/JFH1 chimeric HCV. (A) Gene structures of the flavivirus and hepacivirus and the experimental procedure. Intracellular HCV RNA (B) and extracellular infectious titers (C) were determined at 72-h post-infection with Con1/JFH1 chimeric HCV at an MOI of 1 by qRT-PCR and focus-forming assay, respectively. In all cases, asterisks indicate significant differences (* *p* < 0.01) versus the results of the control cells.
(TIF)

S8 Fig NS1 expression exhibits no effect on viral entry and replication of HCV RNA. (A) BE-KO cells expressing either ApoE or HA-tagged NS1 (HA-NS1) were inoculated with pseudotype particles bearing HCV envelope proteins E1 and E2 (HCVpp) (left) or VSV-G protein (VSVpp) (right), and luciferase activity was determined at 48-h post-infection. (B) Subgenomic HCV RNA replicon of the JFH1 strain was electroporated into the BE-KO cells with/without expression of ApoE or HA-NS1 by the lentiviral vectors, and the remaining colonies were fixed with 4% paraformaldehyde and stained with crystal violet at 1-month post-electroporation after selection with 1 mg/ml of G418.
(TIF)

S9 Fig ApoE has no influence on the physical features of pestivirus. (A) Experimental procedure. (B) The SK6 cells expressing either HA-tagged E^rns^ (HA-E^rns^, middle) or HA-ApoE (bottom) were electroporated with pestivirus RNA. At 72-h post-electroporation, the culture supernatants were subjected to density gradient fractionation, and viral RNA copies (Left panels) and infectious titers (Right panels) for each fraction were determined.
(TIF)

S10 Fig NS1, E^rns^ and apolipoproteins play a common role in the infectious particle formation of *Flaviviridae* viruses. (TIF)
