# Supplementary figures and images for: Host-derived apolipoproteins play comparable roles with viral secretory proteins Erns and NS1 in the infectious particle formation of Flaviviridae
Source: PLoS Pathog. 2017 Jun 23;13(6):e1006475. doi: 10.1371/journal.ppat.1006475 (PMC5500379; doi:10.1371/journal.ppat.1006475)

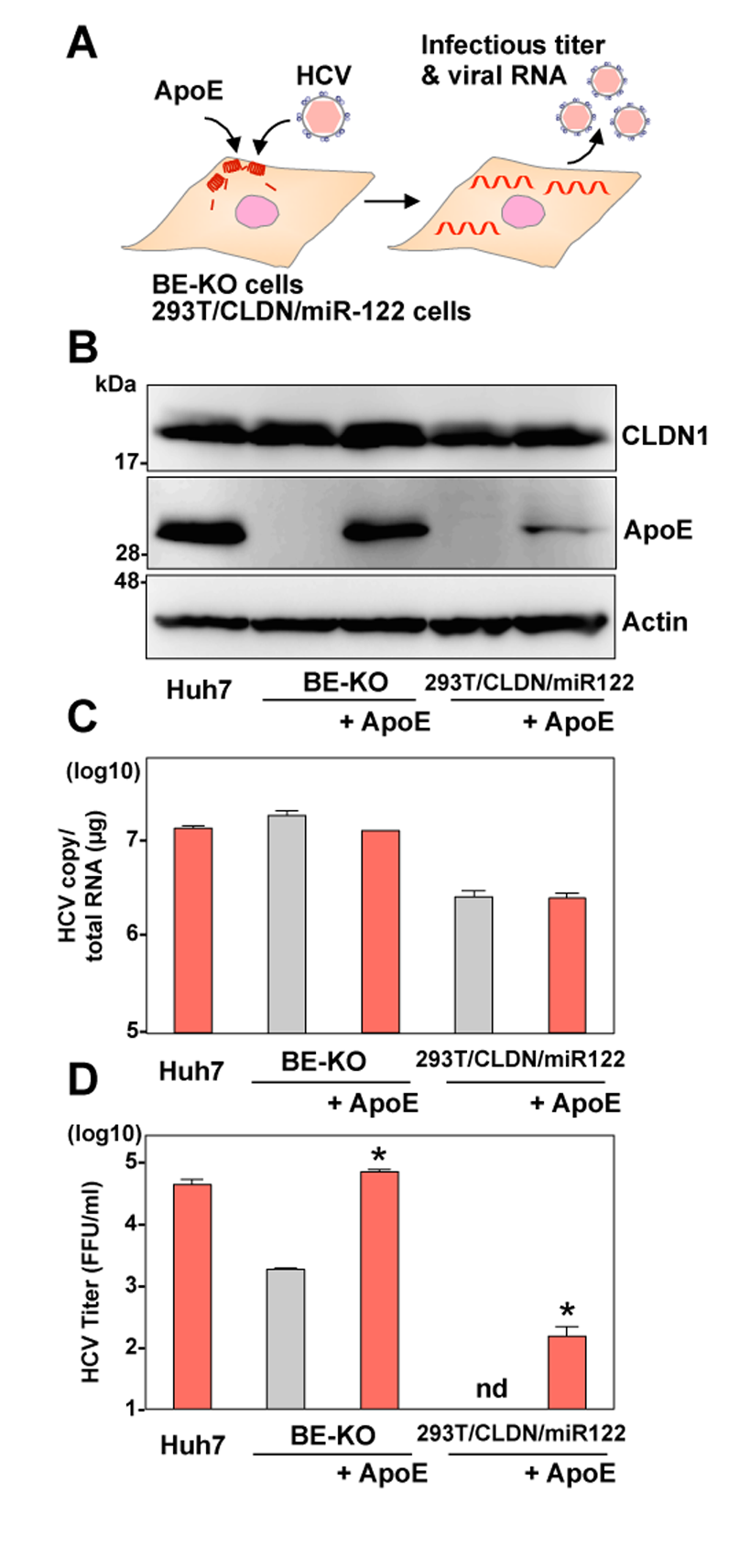

Supplement: S1 Fig — (A) Experimental procedure. (B) Expression of CLDN1 and ApoE was determined by immunoblotting at 48-h post-transduction of lentiviruses into BE-KO and 293T cells. Intracellular HCV RNA (C) and extracellular infectious titers (D) were determined at 72-h post-infection with JFH1 HCV at an MOI of 1 (BE-KO cells) and 10 (293T cells) by qRT-PCR and focus-forming assay, respectively. In all cases, asterisks indicate significant differences (* p < 0.01) versus the results of the control cells. (TIF) [file ppat.1006475.s001.tif]

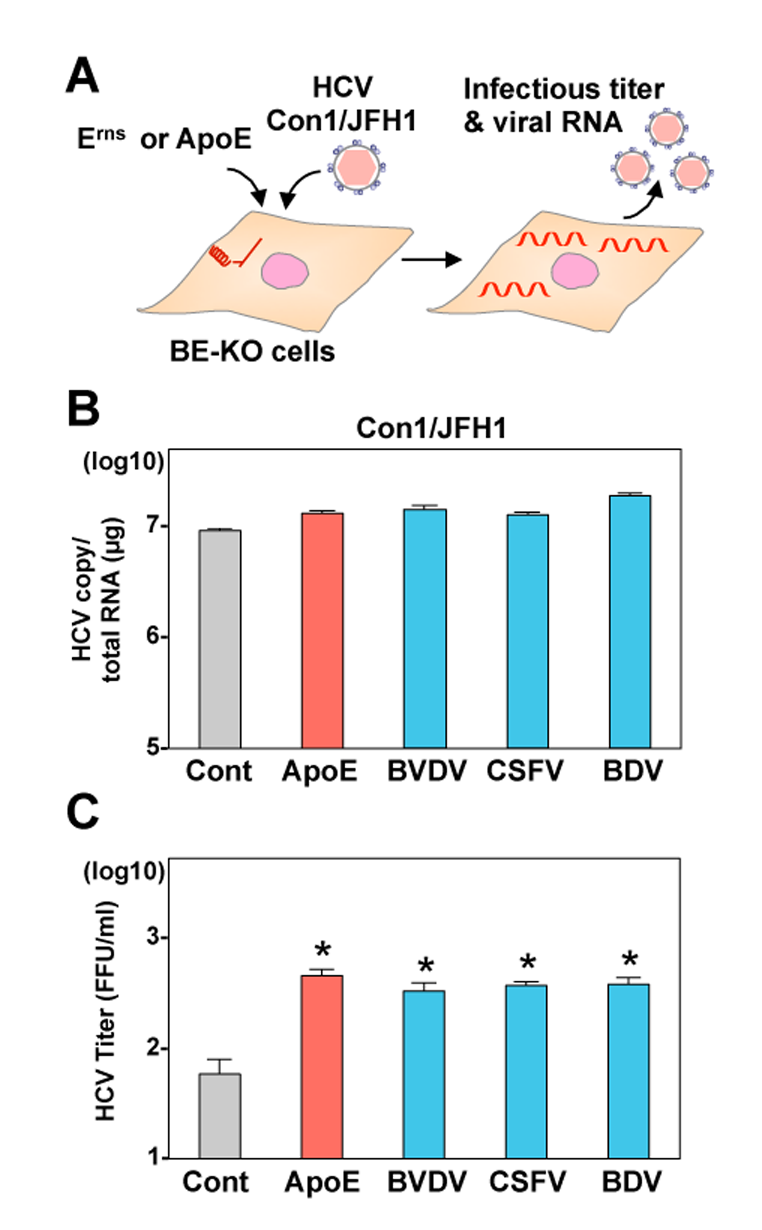

Supplement: S2 Fig — (A) Experimental procedure. Intracellular HCV RNA (B) and extracellular infectious titers (C) in BE-KO cells at 48-h post-transduction with lentiviruses were determined at 72-h post-infection with Con1/JFH1 chimeric HCV at an MOI of 1 by qRT-PCR and focus-forming assay, respectively. In all cases, asterisks indicate significant differences (* p < 0.01) versus the results of the control cells. (TIF) [file ppat.1006475.s002.tif]

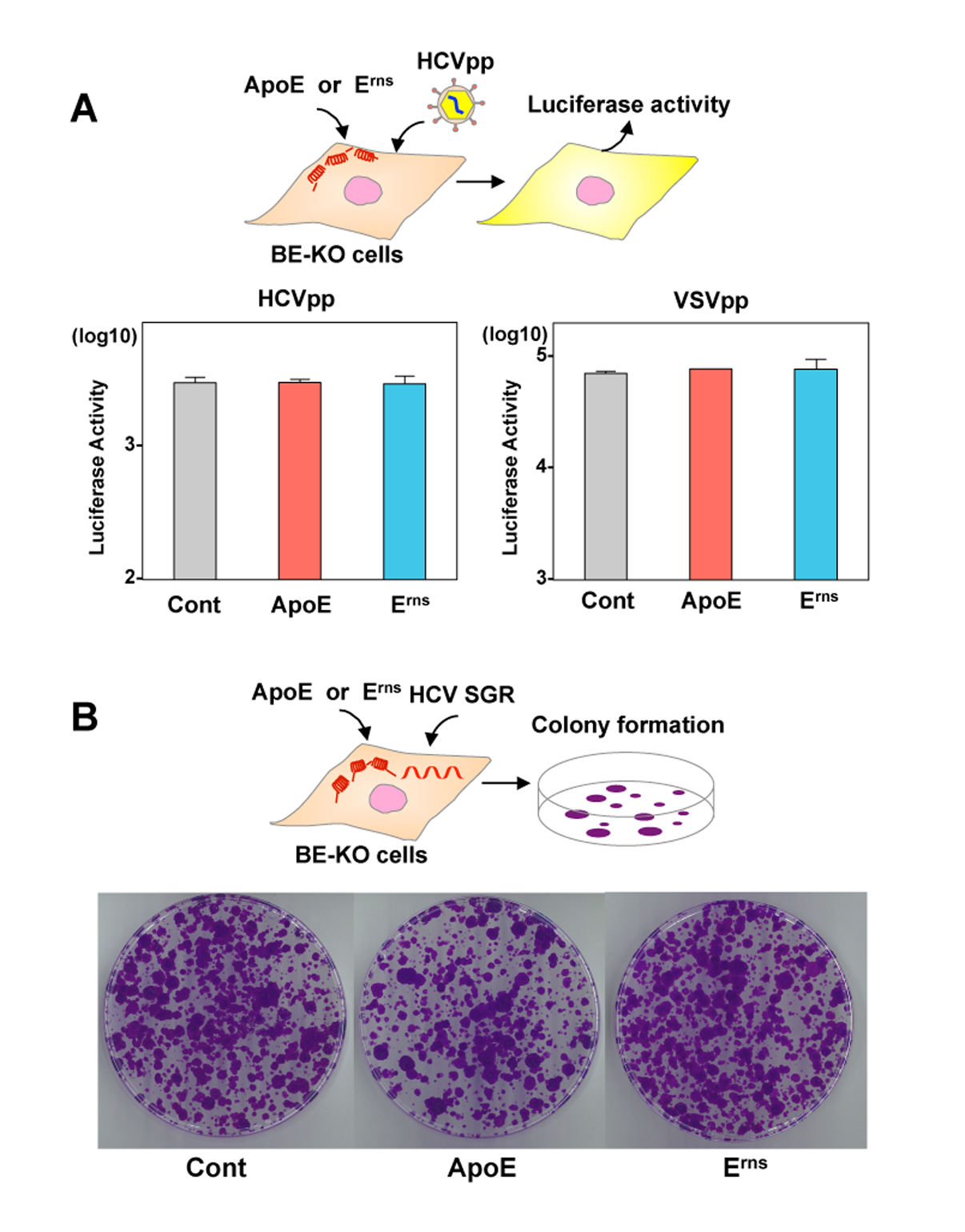

Supplement: S3 Fig — (A) BE-KO cells expressing either ApoE or HA-tagged Erns (HA-Erns) were inoculated with pseudotype particles bearing HCV envelope proteins E1 and E2 (HCVpp) (left) or VSV-G protein (VSVpp) (right), and luciferase activity was determined at 48-h post-infection. (B) Subgenomic HCV replicon RNA of the JFH1 strain was electroporated into the BE-KO cells with/without expression of ApoE or HA-Erns by the lentiviral vectors, and the remaining colonies were fixed with 4% paraformaldehyde and stained with crystal violet at 1-month post-electroporation after selection with 1 mg/ml of G418. (TIF) [file ppat.1006475.s003.tif]

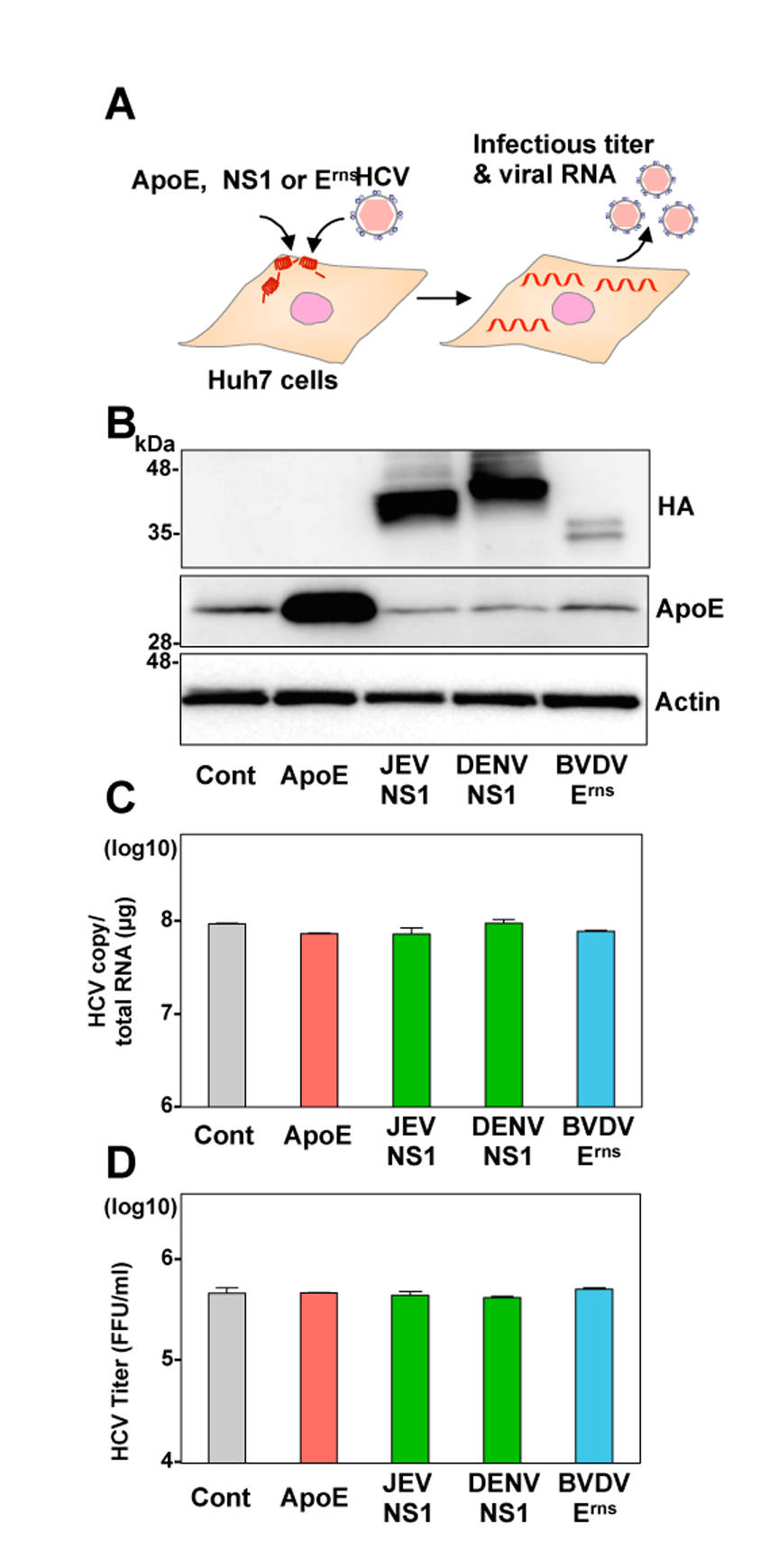

Supplement: S4 Fig — (A) Experimental procedure. (B) Expression of ApoE, HA-tagged NS1 (HA-NS1) from JEV and DENV, and HA-Erns from BVDV was determined by immunoblotting at 48-h post-transduction of lentiviruses into the BE-KO cells. Intracellular HCV RNA (C) and extracellular infectious titers (D) were determined at 72-h post-infection with JFH1 HCV at an MOI of 1 by qRT-PCR and focus-forming assay, respectively. (TIF) [file ppat.1006475.s004.tif]

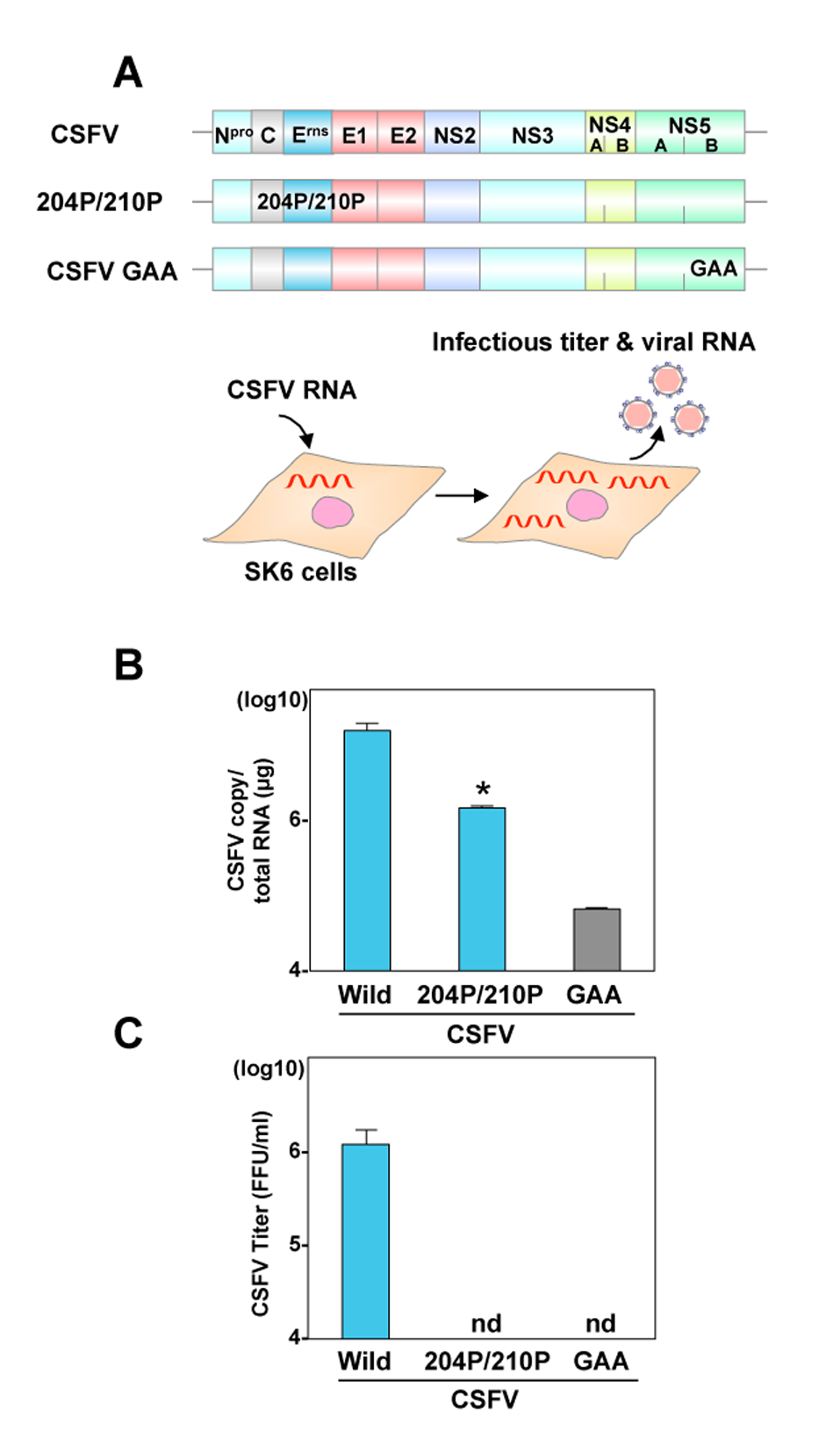

Supplement: S5 Fig — (A) Schematics of CSFV of the wild type and mutants possessing insertion of proline (204P/210P) in Erns and replacement in polymerase dead (GAA), and the experimental procedure. (B) Intracellular CSFV RNA and extracellular infectious titers (C) were determined at 72-h post-electroporation with CSFV RNA by qRT-PCR and focus-forming assay, respectively. (TIF) [file ppat.1006475.s005.tif]

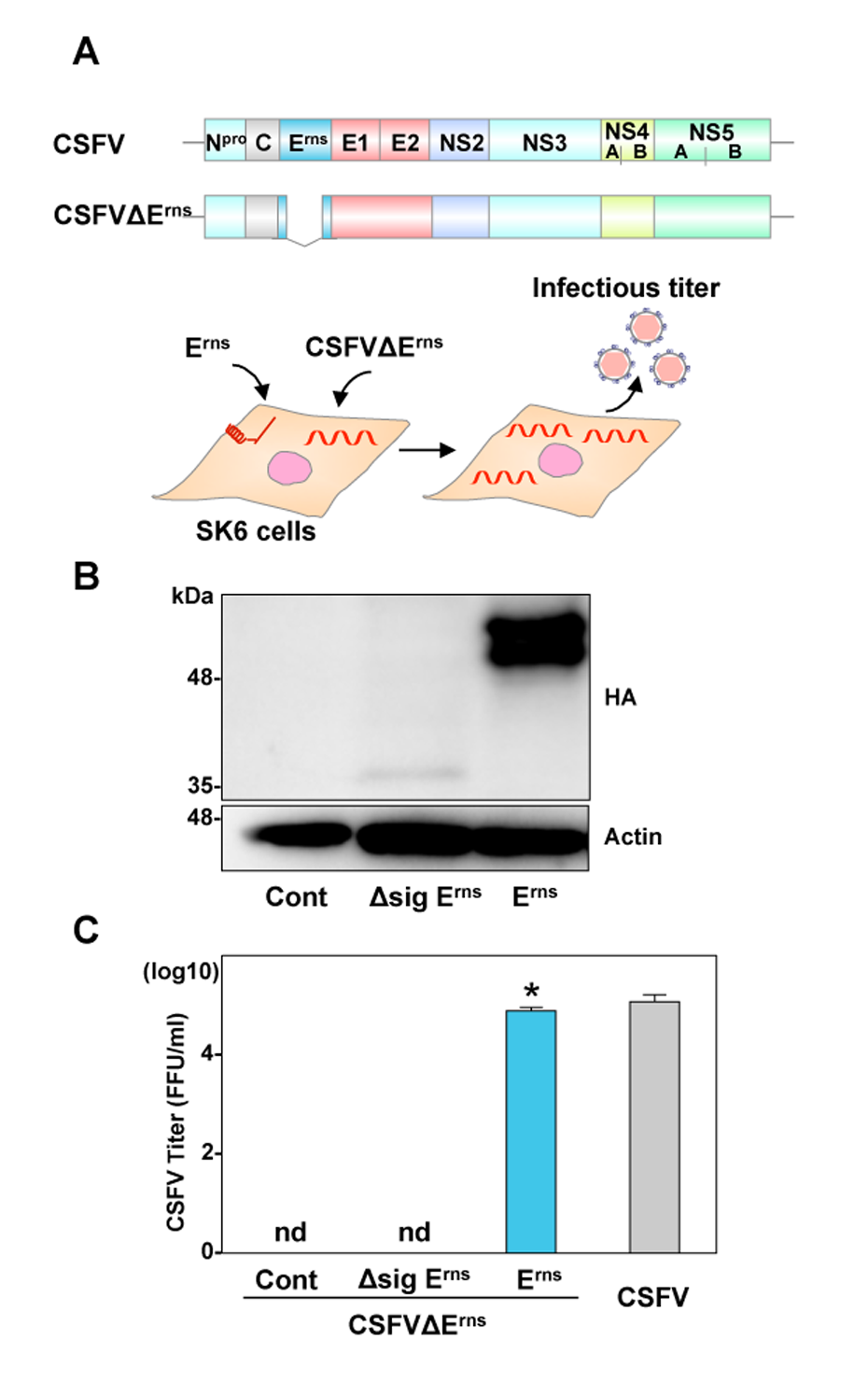

Supplement: S6 Fig — (A) Schematics of the wild type and Erns deletion (CSFVΔErns) RNA, and the experimental procedure. (B) Expression of HA-tagged Erns (HA-Erns) with or without the signal sequence of the core protein (Erns or Δsig Erns) was determined by immunoblotting at 48-h post-transduction of lentiviruses into SK6 cells. (C) Extracellular infectious titer in cells expressing either HA-Erns or HA-Δsig Erns was determined at 72-h post-electroporation with CSFVΔErns by focus-forming assay. (TIF) [file ppat.1006475.s006.tif]

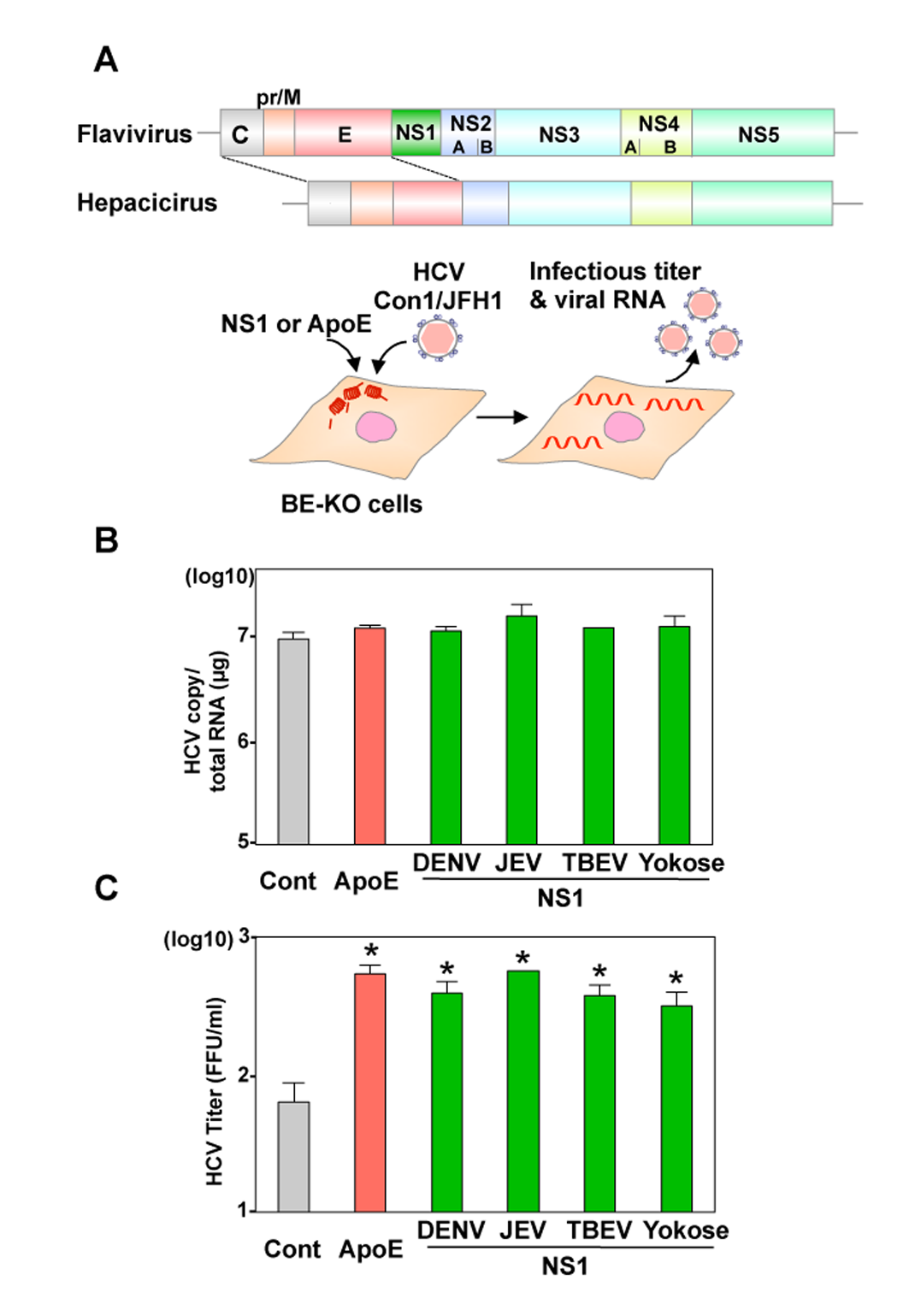

Supplement: S7 Fig — (A) Gene structures of the flavivirus and hepacivirus and the experimental procedure. Intracellular HCV RNA (B) and extracellular infectious titers (C) were determined at 72-h post-infection with Con1/JFH1 chimeric HCV at an MOI of 1 by qRT-PCR and focus-forming assay, respectively. In all cases, asterisks indicate significant differences (* p < 0.01) versus the results of the control cells. (TIF) [file ppat.1006475.s007.tif]

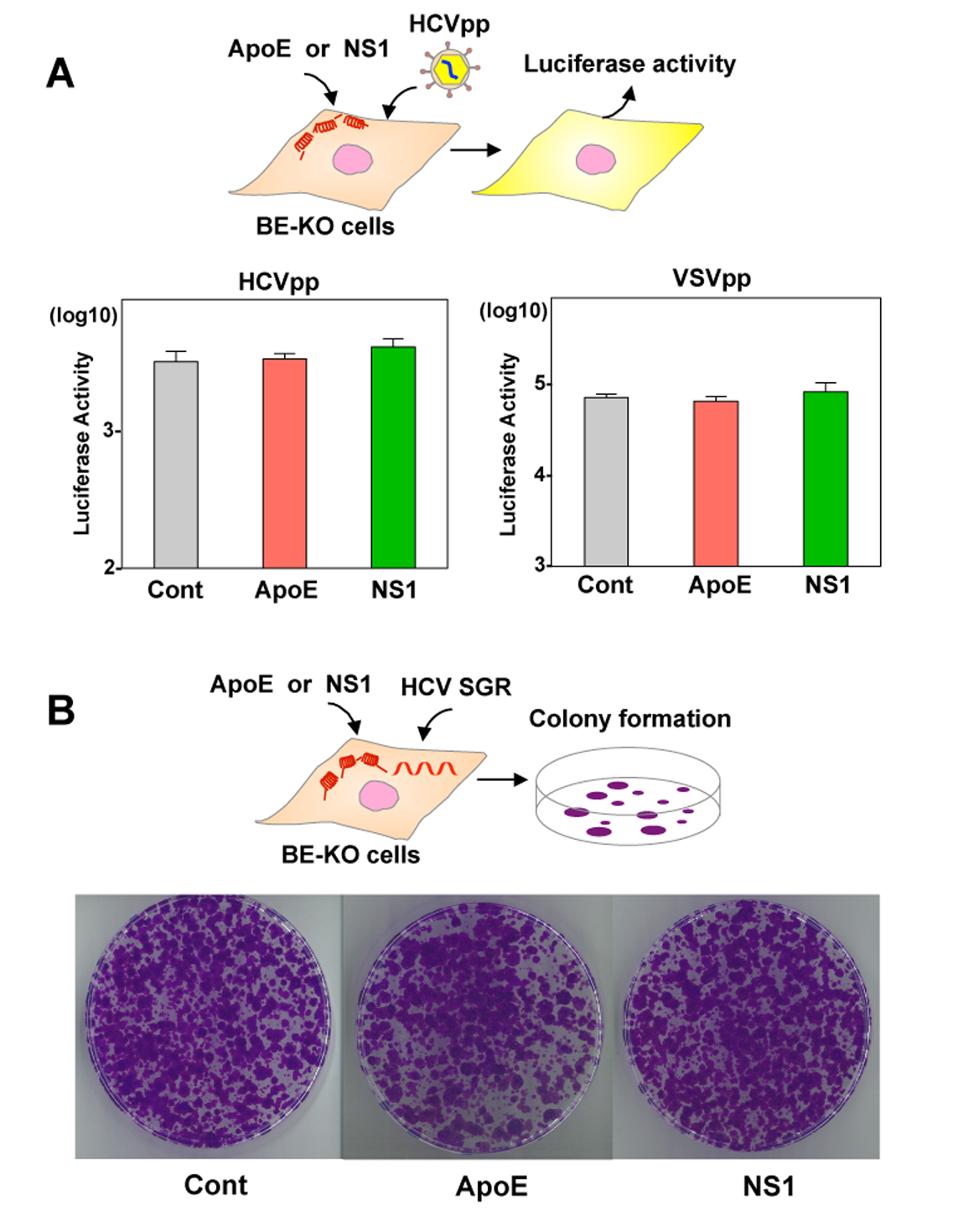

Supplement: S8 Fig — (A) BE-KO cells expressing either ApoE or HA-tagged NS1 (HA-NS1) were inoculated with pseudotype particles bearing HCV envelope proteins E1 and E2 (HCVpp) (left) or VSV-G protein (VSVpp) (right), and luciferase activity was determined at 48-h post-infection. (B) Subgenomic HCV RNA replicon of the JFH1 strain was electroporated into the BE-KO cells with/without expression of ApoE or HA-NS1 by the lentiviral vectors, and the remaining colonies were fixed with 4% paraformaldehyde and stained with crystal violet at 1-month post-electroporation after selection with 1 mg/ml of G418. (TIF) [file ppat.1006475.s008.tif]

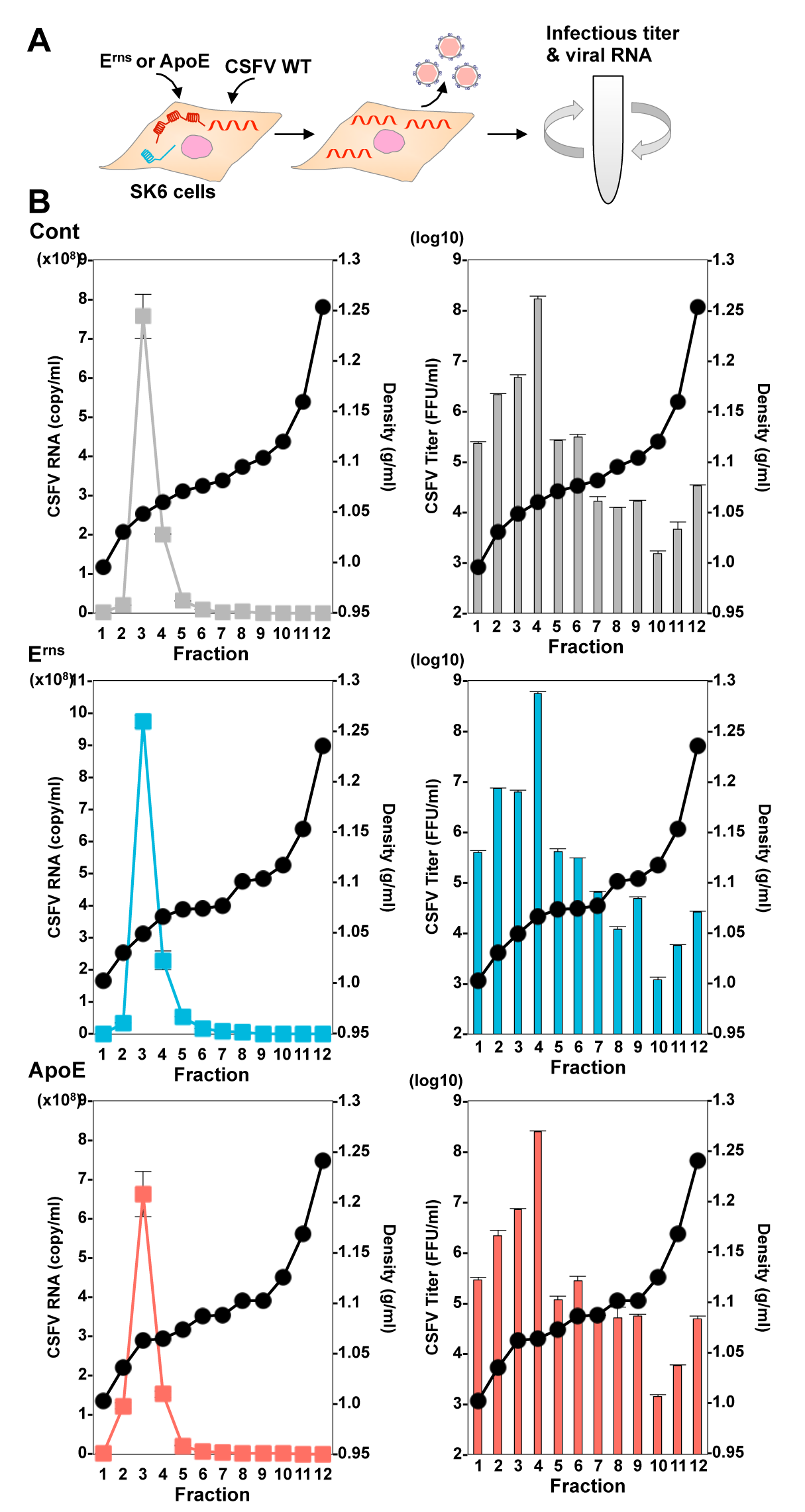

Supplement: S9 Fig — (A) Experimental procedure. (B) The SK6 cells expressing either HA-tagged Erns (HA-Erns, middle) or HA-ApoE (bottom) were electroporated with pestivirus RNA. At 72-h post-electroporation, the culture supernatants were subjected to density gradient fractionation, and viral RNA copies (Left panels) and infectious titers (Right panels) for each fraction were determined. (TIF) [file ppat.1006475.s009.tif]

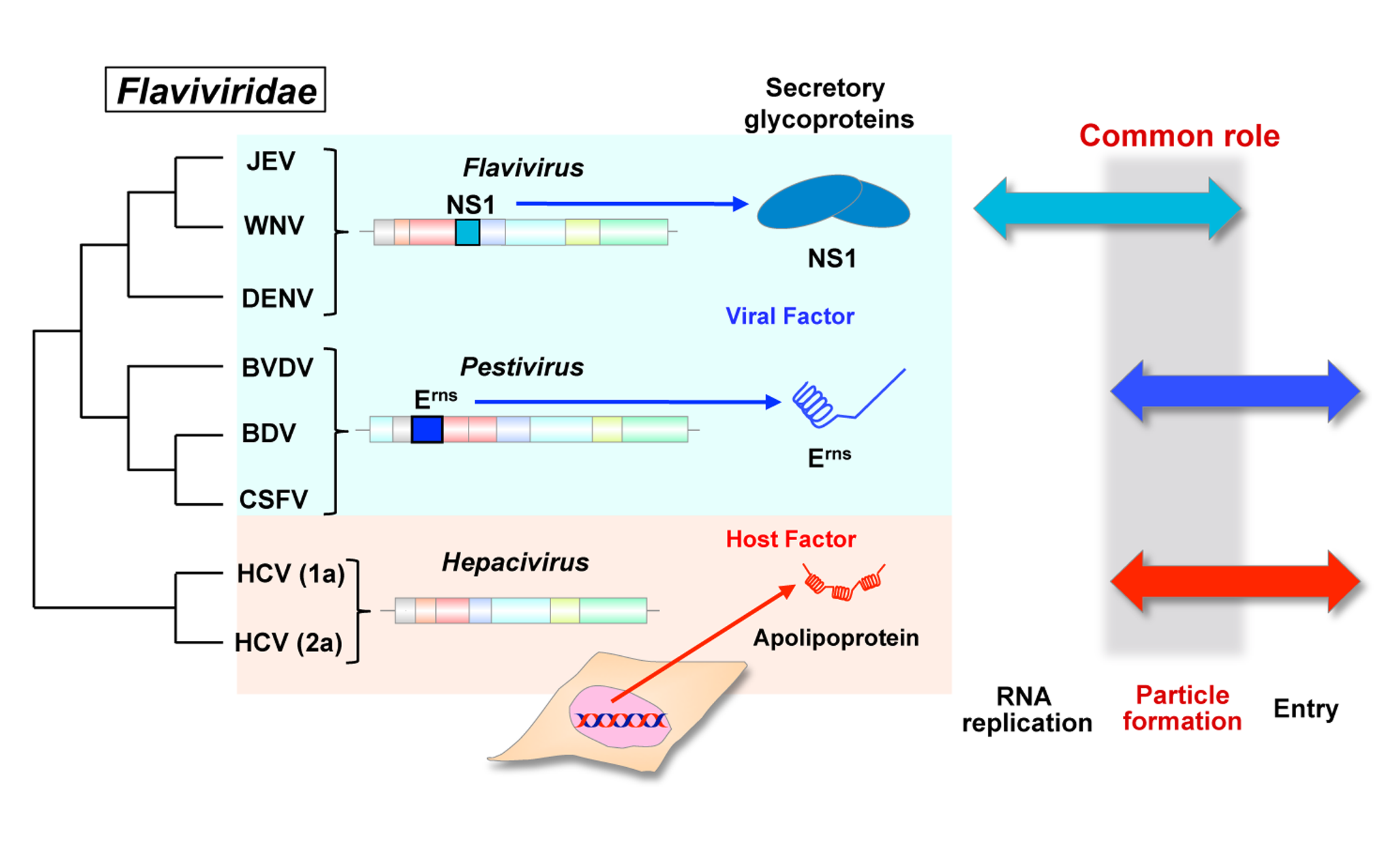

Supplement: S10 Fig — (TIF) [file ppat.1006475.s010.tif]
